# Systematic review of thyroid function in *NKX2-1*-related disorders: Screening and diagnosis

**DOI:** 10.1371/journal.pone.0303880

**Published:** 2024-07-11

**Authors:** Beatriz Carmona-Hidalgo, Carmen Martín-Gómez, Estefanía Herrera-Ramos, Rocío Rodríguez-López, Laia-Nou Fontanet, José C. Moreno, Juan Antonio Blasco-Amaro, Juliane Léger, Juan Dario-Ortigoza-Escobar, NKX2-1-Related Disorders Guideline Working Group

**Affiliations:** 1 Health Technology Assessment Area-AETSA, Andalusian Public Foundation for Progress and Health (“Fundación Progreso y Salud”–“FPS”), Seville, Spain; 2 Research Group HUM604: Lifestyle Development in the Life Cycle and Health Promotion, University of Huelva, Huelva, Spain; 3 Evaluation Unit of the Canary Islands Health Service (SESCS), Canary Islands Health Research Institute Foundation (FIISC), Santa Cruz of Tenerife, Spain; 4 Department of Child Neurology, Movement Disorders Unit, Institut de Recerca Sant Joan de Déu, Barcelona, Spain; 5 Thyroid Molecular Laboratory, Institute for Medical and Molecular Genetics (INGEMM). Research Institute of Paz University Hospital (IdiPAZ), Madrid, Spain; 6 U-753 The Rare Diseases Networking Biomedical Research Centre (CIBERER), Instituto de Salud Carlos III, Madrid, Spain; 7 European Reference Network on Rare Endocrine Conditions (Endo-ERN), Amsterdam, The Netherlands; 8 Endocrinology-Diabetology Department, Assistance Publique-Hôpitaux de Paris, Robert Debre´ University Hospital, Reference Center for Growth and Development Endocrine Diseases, Paris, France; 9 Université Paris Cité, NeuroDiderot, Institut National de la Santé et de la Recherche Médicale (INSERM 1141), Paris, France; 10 U-703 Centre for Biomedical Research on Rare Diseases (CIBER-ER), Instituto de Salud Carlos III, Barcelona, Spain; 11 European Reference Network for Rare Neurological Diseases (ERN-RND), Tübingen, Germany; Central South University, CHINA

## Abstract

**Background:**

*NKX2-1*-related disorders (*NKX2-1*-RD) are rare conditions affecting lung, thyroid, and brain development, primarily caused by pathogenic variants or deletions in the *NKX2-1* gene. Congenital hypothyroidism (CH) is a common endocrine manifestation, leading to irreversible intellectual disability if left untreated.

**Objectives:**

The aim was to evaluate the current evidence for the use of screening and diagnostic techniques for endocrine alterations in patients with *NKX2-1*-RD.

**Methods:**

This systematic review was reported following the PRISMA guidelines. Two separate research questions in PICO format were addressed to cover initial screening and diagnosis procedures for endocrine diseases in patients with *NKX2-1*-RD. Eligibility criteria focused on patients with genetic confirmation of the disease and hypothyroidism. Various databases were searched, and data were extracted and assessed independently by two reviewers.

**Results:**

Out of 1012 potentially relevant studies, 46 were included, for a total of 113 patients. CH was the most frequent endocrine alteration (45% of patients). Neonatal screening was reported in only 21% of patients based on blood TSH measurements. TSH thresholds varied widely across studies, making hypothyroidism detection ranges difficult to establish. Diagnostic tests using serum TSH were used to diagnose hypothyroidism or confirm its presence. 35% of patients were diagnosed at neonatal age, and 42% at adult age. Other hormonal dysfunctions identified due to clinical signs, such as anterior pituitary deficiencies, were detected later in life. Thyroid scintigraphy and ultrasonography allowed for the description of the thyroid gland in 30% of cases of hypothyroidism. Phenotypic variability was observed in individuals with the same variants, making genotype-phenotype correlations challenging.

**Conclusion:**

This review highlights the need for standardized protocols in endocrine screening for *NKX2-1*-RD, emphasizing the importance of consistent methodology and hormone threshold levels. Variability in *NKX2-1* gene variants further complicates diagnostic efforts. Future research should concentrate on optimizing early screening protocols and diagnostic strategies.

## Introduction

*NKX2-1*-related disorders (*NKX2-1*-RD, OMIM#610978), also known as Benign Hereditary Chorea (BHC), is a rare condition that affects the development and function of the lungs, thyroid, and brain. It is caused by variants or deletions in the NK2 homeobox 1 gene (*NKX2-1*, OMIM*600635), although other mechanisms have been described, including deletions outside the *NKX2-1* gene [1–3] and Alu retrotransposition events [4] that explain some additional cases. This allelic variability can contribute to explaining the phenotypic variability observed in patients. *NKX2-1* regulates the expression of specific genes that are essential for the development and maturation of these organs. A subgroup of individuals affected by *NKX2-1*-RD may develop neonatal respiratory distress syndrome and/or congenital hypothyroidism (CH). From a neurological standpoint, it can also cause hypotonia, motor delay, and movement disorders [3, 5]. *NKX2-1*, *PAX8*, *FOXE1*, and *HHEX* are among the transcription factors that are co-expressed in follicular and thyroid precursor cells during the early stages of embryonic development. Survival and differentiation of thyroid precursor cell are dependent on *NKX2-1*. In the absence of *NKX2-1*, the regulatory network is disrupted, which results in precursor cells apoptosis and impaired thyroid gland development. Germline and somatic pathogenic variants of the *NKX2-1* gene disturb its ability to bind to the target DNA, resulting in the loss of its regulatory function and potentially facilitating malignant transformation [6, 7].

In infants, CH is the most prevalent endocrine disease, affecting between 1 in 2000 and 4000 newborns. In 65% of cases, CH is associated with developmental abnormalities of the thyroid gland (thyroid dysgenesis). In these cases, the thyroid gland may be absent (agenesis, 30%), ectopically located (60%), and/or severely reduced in size (hypoplasia, 10%). In the remaining 35% of cases, CH is associated with gland in situ, of which 50% with goiter, due to deficiencies in thyroid hormone biosynthesis (dyshormonogenesis) [8, 9]. A diagnosis of CH is established in the presence of elevated serum thyroid-stimulating hormone (TSH) and low total thyroxine (T4) or free T4 (fT4) levels. In contrast to normal serum T4 levels, subclinical or compensated hypothyroidism is characterized by marginally increased TSH levels in the blood. Considering that untreated CH during infancy can result in irreversible intellectual disability and physical development delay, early screening is essential for identifying affected individuals [10].

Thyroid function tests added to neonatal screening (NBS) programs began to be performed in the 1970s, reducing the neurological developmental impairment caused by the disease. NBS for CH is routine in most countries worldwide. Currently, most NBS around the world employ a primary TSH strategy. However, certain state programs, as exemplified in Japan and the Netherlands, deviate from this approach by including measurements of T4 in their NBS protocols [11]. Neonates suspected of having CH are identified based on a country-specific TSH threshold [12]. However, screening method, age at specimen collection, prematurity status, and newborn clinical factors affect threshold selection and interpretation. This threshold is important because it determines which infants will undergo CH confirmation testing [13, 14]. These newborns can begin treatment within the first weeks of life to rapidly normalize thyroid hormone levels [15]. Lifelong monitoring of serum TSH and T4 levels guarantees optimal thyroid hormone levels and a favorable prognosis [8, 15, 16], which encompasses improved neurodevelopmental outcomes [17]. Thyroid ultrasonography and scintigraphy, assist in identifying the location and echogenicity of the thyroid gland, but treatment can be initiated without these tests. Considering the small number of cases, comprehensive studies and systematic reviews are necessary to gain insight into the screening and diagnostic techniques used for endocrine changes in patients with *NKX2-1*-RD.

The European Reference Network for Rare Neurological Disorders (ERN-RND) and the European Network for Rare Endocrine Conditions (Endo-ERN) endeavor to establish the initial clinical practice guidelines for *NKX2-1*-RD as a part of the European Program ERN Guidelines. Hence, the objective of this systematic review is to provide an exhaustive evaluation of the screening and diagnostic methodologies employed to detect endocrine modifications in individuals afflicted with *NKX2-1*-RD.

## Methods

This study comprises the systematic reviews of two research questions reported following the Preferred Reporting Items for Systematic Review and Meta-Analyses (PRISMA) statement [18] **(**S1 File**)**.

### Research questions

They were translated into PICO format (acronym for Population-Intervention-Comparator-Outcome) to guide the literature search. The main PICO question addressed was: *What sort of endocrinologic follow-up is recommended to monitor the onset of endocrinologic diseases in NKX2-1-related disorders*?. It was divided into three research questions to cover all the aspects related to the detection, diagnosis, treatment, and follow-up of endocrine alterations in *NKX2-1-*RD. An ongoing study will address treatment and follow-up.

In the present study, we focus on the research questions about detection and diagnosis: *1) What are the best procedures for initial screening of endocrine diseases in patients with NKX2-1-related disorders*?, and *2) What are the best procedures for diagnosis of endocrine diseases in patients with NKX2-1-related disorders*?. The protocols of the systematic reviews were previously registered in the International Prospective Register of Systematic Reviews (PROSPERO) repository with the identifications, CRD42022340996 and CRD42022341002, respectively.

### Eligibility criteria

Specific inclusion criteria were employed to select the relevant studies for the systematic reviews. The population criteria were common for both research questions: patients of all ages with genetic confirmation of the disease (pathogenic variants in *NKX2-1*, previously known as TTF-1, or deletion in the 14q13.3 chromosome) with hypothyroidism. Non-human studies and patients without genetic confirmation of the disease were excluded. Given the rare condition, any comparator was used. The interventions and outcomes were specific to each research question:

1. *What are the best procedures for initial screening of endocrine diseases in patients with NKX2-1-related disorders*? Interventions: they were divided into antenatal screening (fetus ultrasound examination, cordocentesis, fetal blood sampling, and family inheritance of TTF-1/NKX2-1 mutation or deletion) and/or neonatal screening (thyroid hormone levels in blood spots and hearing tests). Outcomes: detection of suspicion of hypothyroidism, thyroid dyshormonogenesis and/or dysgenesis, neurodevelopmental alterations, and congenital malformations in the newborn.
2. *What are the best procedures for the diagnosis of endocrine diseases in patients with NKX2-1-related disorders*? Interventions: quantification of TSH and fT4 levels in serum and thyroid gland imaging tests (scintigraphy and/or ultrasonography). Outcomes: thyroid hormone levels in serum to confirm the presence of hypothyroidism and thyroid gland volume and location.

Interventions related to hypothyroidism but not to *NKX2-1-*RD were excluded. The included study designs were primary studies, systematic reviews, and randomized controlled trials (RCTs). Narrative reviews, protocols, conference articles, editorials, letters to the editor, and those whose full text was unavailable to be retrieved were excluded.

### Search strategy

A systematic literature search was carried out for each research question to review the scientific evidence. PubMed, Embase, the Cochrane Library, and MEDLINE (Ovid) were searched as reference databases. Other resources were APA PsyncINFO, CINAHL Complete, the TRIP medical database, and ICTPR. Subject-specific databases were included to retrieve information in rare disease resources such as Orphanet, EURORDIS, NORD, RARE-Best Practices, and Gene Reviews. Finally, the International Health Technology Assessment (HTA) database, a systematic review-specific resource, was included for the retrieval of technology assessments.

The search covered the period from January 2002 to May 2022, and it was updated in June 2023. The included records date back to 2002, when pathogenic variants in the *NKX2-1* gene were first associated with benign hereditary chorea [19]. Both controlled language (descriptors) and free terminology were used to search for studies. The initial strategies were carried out in MEDLINE (Ovid) and later adapted to each database’s syntax. The original search strategies are specified in S2 File.

### Study screening and data extraction

The identified references were imported into the reference management section of the software application Covidence (https://www.covidence.org/), and the duplicates were removed. A different review was created for each question. Two authors independently filtered the references by title, abstract, and full text (BCH and CMG for the screening research question and BCH and JDOE for the diagnosis research question) according to the inclusion criteria. The disagreements between the reviewers were resolved by a third researcher, (JL). The data were extracted by two independent authors (BCH and JDOE) and recorded in Excel spreadsheets. Specific details of the studies (author, year of publication, study type, and location) and specific clinical data of each patient (age, sex, genetic tests, thyroid, neurological, and respiratory alterations, age at onset of hypothyroidism, screening and diagnosis methods, hormone levels, and thyroid gland volume and/or location alteration) were extracted.

### Quality assessment

The quality of the studies was assessed independently by two authors (BCH and JDOE). Disagreements were resolved through discussion to reach a consensus. The quality assessment was carried out using specific tools for each type of study. The included references were classified as case reports, case series, and cohort studies. The tool developed by Murad et al. [20], used for case reports and case series, allows the evaluation of the methodological quality of studies with a low body of evidence but is essential for the decision-making process. It uses eight items categorized into four domains (selection, ascertainment, causality, and reporting). According to the criteria of the tool, the quality was rated by summing the scores of the eight binary responses (1 = low risk of bias, 0 = high risk of bias) into an aggregate score. The score ranges from 0 (poor quality) to 8 (high quality) [21]. The items of ascertainment, causality, and reporting were adapted to the specific clinical scenario included in the research questions. Regarding cohort studies, we used the Newcastle-Ottawa Scale (NOS), which can also be modified based on a special subject [22]. This tool uses three categories (selection, exposure, and comparability) with eight items to identify good-quality decisions using starts. One star can be given to each item in the selection (maximum of 4 stars) and outcome (maximum of 3 stars) categories, and a maximum of two stars for comparability. No studies were excluded based on low-quality grounds. The Grade of Recommendation, Assessment, Development, and Evaluation System (GRADE) was established to evaluate the quality of each outcome provided by the evidence of the studies. This system sets four categories (high, moderate, low, and very low) with five factors: risk of bias, inconsistency, indirectness, imprecision, and publication bias [23].

## Results

### Study selection

**Fig 1:**
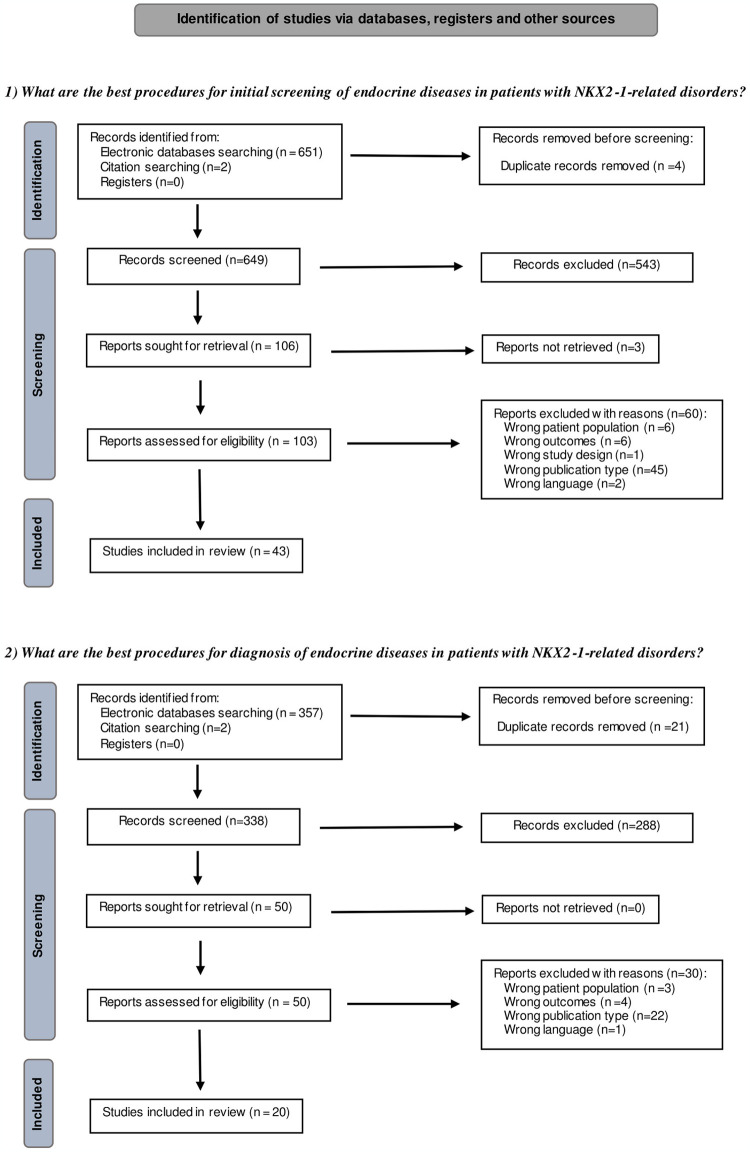
PRISMA flowchart. Illustration of the PRISMA flowchart depicting the title-abstract and full-text screening of the articles related to the detection and diagnosis of endocrine diseases in patients with *NKX2-1-*RD.

During the initial search, a total of 1012 records were located. After removing duplicates, 987 potentially relevant studies remained (649 in the first research question and 338 in the second one). Title and abstract screening was carried out, yielding 106 and 50 studies, respectively. According to inclusion and exclusion criteria, 63 studies were included following full-text screening for both questions. After removing common studies between both research questions, 46 studies were finally analyzed for this study. The screening processes (PRISMA flowcharts) [24] are depicted in Fig 1. The included and excluded articles after full-text review, along with the reasons for their exclusion, are included in S3 File. The excluded studies were classified according to the first criterion that did not fit the PICO format.

### Study characteristics

52% of the included studies were case series (n = 24), 43% were case reports (n = 20), and 5% were cohorts (n = 2). Due to the rare clinical condition studied, no anticipated randomized controlled trial or systematic review was available. 63% of the data originated from Europe (n = 29), 22% from Asia (n = 10), 13% from America (n = 6), and 2% from Australia (n = 1) (Table 1).

**Table 1:** Presents a comprehensive list of references that were included in this study for the purpose of investigating the initial screening (detection) and diagnosis of thyroid diseases in individuals affected by *NKX2-1*-RD. The author, year of publication, location, and the number of patients included in each study for screening and/or diagnosis are specified.

Author, year	Location	Study type	Screening	Diagnosis	Author, year	Location	Study type	Screening	Diagnosis
**Balicza, 2018**	Hungary	Case series	**1**	**1**	**Monti, 2015**	Italy	Case report	**1**	**0**
**Barnett, 2012**	Australia	Case report	**1**	**1**	**Moya, 2006**	Spain	Case series	**3**	**0**
**Barreiro, 2011**	Spain	Case report	**1**	**1**	**Moya, 2018**	Spain	Case report	**1**	**0**
**Carré, 2009**	France	Case series	**6**	**0**	**Nakamura, 2012**	Japan	Case series	**3**	**0**
**Delestrain, 2023**	France	Case series	**1**	**0**	**Narumi, 2010**	Japan	Cohort	**1**	**0**
**De Filippis, 2014**	Italy	Case series	**2**	**0**	**Nattes, 2017**	France	Case series	**9**	**9**
**Doyle, 2004**	United States	Case series	**4**	**4**	**Parnes, 2019**	United States	Case series	**4**	**0**
**Ferrara, 2008**	Italy	Case series	**3**	**0**	**Peall, 2014**	United Kingdom	Case series	**4**	**0**
**Gentile, 2016**	Italy	Case Report	**0**	**1**	**Prasad, 2019**	United Kingdom	Case report	**1**	**1**
**Gillett, 2013**	United States	Case report	**1**	**0**	**Salerno, 2014**	Italy	Case report	**1**	**1**
**Gras, 2012**	France	Case series	**18**	**0**	**Salvado, 2013**	Spain	Case series	**3**	**3**
**Hayasaka, 2018**	Japan	Case series	**3**	**0**	**Salvatore, 2010**	Italy	Case series	**3**	**3**
**Hayashi, 2015**	Japan	Case series	**2**	**0**	**Santos-Silva, 2019**	Portugal	Case report	**1**	**1**
**Hermanns, 2018**	Germany	Case report	**1**	**0**	**Shiohama, 2018**	Japan	Case report	**1**	**0**
**Kharbanda, 2017**	United Kingdom	Case report	**1**	**1**	**Tanaka, 2020**	Japan	Case report	**1**	**1**
**Kleinlein, 2011**	Germany	Case report	**1**	**0**	**Tozawa, 2016**	Japan	Case report	**1**	**0**
**Koht, 2016**	Norway	Case series	**5**	**0**	**Trevisani, 2022**	Italy	Case report	**0**	**1**
**Krude, 2002**	Germany	Case series	**5**	**0**	**Uematsu, 2012**	Japan	Case series	**3**	**3**
**Li, 2023**	China	Case series	**1**	**1**	**Veneziano, 2014**	United Kingdom	Case series	**0**	**2**
**Lynn, 2020**	United States	Case report	**1**	**1**	**Villafuerte, 2018**	Spain	Case report	**1**	**0**
**Magrinelli, 2022**	United Kingdom	Case series	**1**	**0**	**Villamil-Osorio, 2021**	Colombia	Case report	**1**	**0**
**Makretskaya, 2018**	Russia	Cohort	**2**	**2**	**Williamson, 2014**	United Kingdom	Case series	**3**	**0**
**Maquet, 2009**	Switzerland	Case report	**1**	**0**	**Zou, 2018**	Saudi Arabia	Case series	**1**	**0**

### Patients demographics

Only patients diagnosed with *NKX2-1*-RD and hypothyroidism were considered (113 patients). At the time of the study, 1.7% of patients were neonates (≤ 28 days of life) (n = 2), 41.6% were children (≤12 years of age) (n = 47), 14.1% were adolescents (13–19 years of age) (n = 16) and 18.6% were adults (20–65 years old) (n = 21). In 23.9% of patients (n = 27) the age was not reported. The calculated mean age was 15.3 years (range 28 days to 64 years). The gender was quite equally distributed (54% were females) (n = 61). Regarding pathogenic *NKX2-1* gene variants or deletions, there were eight cases (7%) with splicing variants, thirty-three individuals (29%) with missense variants, fifty-three (47%) patients with frameshift variants, eighteen cases (16%) with large deletions, and one patient with an insertion of a 46-bp Alu sequence in the *NKX2-1* gene [4]. One patient carried a double homozygous variant [25] and another two deletions in the short and long arms of chromosome 14 [26]. A patient carried a frameshift variant and a splicing variant [27]. The main technique used to identify pathogenic variants was sequencing in 71.7% of the patients (n = 81). Comparative genomic hybridization (CGH) was used in 10.6% of the cases (n = 12), while Fluorescence in situ Hybridization (FISH) and bacterial artificial chromosomes (BAC) were used in 1.8% of the cases each (n = 2). Next generation sequencing techniques were used in 7% (n = 8), and no diagnostic technique was reported in an additional 7% of patients. In 44 (38.9%) patients, the pathogenic variants were *de novo*. In 37 cases (32.7%), the type of inheritance was not provided.

In our study, CH was the most common endocrine alteration confirmed in 51 patients (45.1%). Compensated or subclinical hypothyroidism was present in twenty-three patients (20.3%), and gestational hypothyroidism during pregnancy was present in three patients (2.7%) [28–30]. The remaining 36 patients in our cohort of 113 presented overt hypothyroidism (31.9%). In addition to hypothyroidism, the presence of other endocrine alterations was found among the patients with NKX2-1-*RD*. A neonatal patient diagnosed with CH demonstrated multiple pituitary hormone deficiencies, including growth hormone and cortisol deficiency. The gonadotrophins were undetectable, and the pituitary gland was small with a normal stalk. Transient hyperinsulinism was present during the neonatal period, requiring a brief period of treatment [26]. The growth hormone deficiency and visual impairment were present in another patient diagnosed with CH [31], and in a patient with subclinical hypothyroidism who showed serum insulin-like growth factor-I deficiency too [32]. Immunodeficiency was significant in a patient diagnosed with primary hypothyroidism in the first year of age [33] and in another patient with subclinical hypothyroidism, fetal growth, a reduction of CD3/CD8, and an increase of the CD4/CD8 ratio [34]. An adult patient was diagnosed with hypothyroidism and another patient with CH during childhood [2], both of them with pituitary disfunction. The low levels of testosterone and luteinizing hormone resulted in hypogonadism that required treatment [35]. Two patients from the same study, diagnosed with mild hypothyroidism at an advanced age, showed a slightly low prolactin level and cystic pituitary mass [36].

Regarding clinical alterations related to *NKX2-1-*RD, 57.8% of patients reported neurological, thyroid, and lung disease (a complete triad). 29.3% of the patients had endocrine and neurological alterations, while only 10% had endocrine and respiratory involvement. Chorea was the most prevalent neurological disorder (88.6% of patients with neurological involvement), followed by gait disorders and neurodevelopmental delay. Neonatal respiratory distress was the most frequent manifestation (45%) in patients with respiratory alterations, followed by respiratory infections and diminished lung capacity. As respiratory alterations are common in premature births, we analyzed whether the presence of these alterations among the patients in our study could be due to early birth or to *NKX2-1-*RD. 62.8% of patients in this study reported respiratory alterations (n = 71), while only 4.2% of them (n = 3) were premature at birth [37, 38]. More details are presented in S4 File.

### Initial screening strategies of endocrine alterations in patients with *NKX2-1*-RD

NBS were conducted utilizing blood spots to measure TSH and fT4 or T4 levels in the first days of life after birth. In this study, the results of the neonatal screening were reported in 21.2% of patients (n = 24) indicating CH in 16.8% (n = 15) of them. The mean and median blood TSH levels were 58.7 mU/L and 44 mU/L, respectively, reported in the 12.4% of patients (n = 14) with neonatal screening. In 9.7% of patients (n = 11), the blood TSH value was reported as normal or high but lacked numerical details. A detail to highlight is that Gillett et al. 2013 [39] reported markedly elevated TSH levels.

No consensus on hormone values for CH detection was found, making it difficult to establish a standard range. Some patients had hormone levels above the normal range, indicating endocrine alteration, but no numbers were given. Eight studies (17.4%) reported a TSH threshold of 10 mU/L or less for neonatal screening to consider CH. Two studies set the threshold to 16.9 mU/L [28, 40]. Other studies did not report cut-off values. All CH confirmatory cases had TSH values above thresholds. Only one study reported fT4 in neonatal screening and TSH in CH patients [41]. Four studies measured T4 [28, 40, 42, 43]. The mean and median (n = 3) were 124.8nmol/L and 151.2 nmol/L. These values were considered normal or low regarding the threshold established by each study. S5 File shows neonatal thyroid hormone levels.

### Diagnosis strategies of endocrine alterations in patients with *NKX2-1*-RD

Diagnostic tests measured the levels of TSH and fT4 or T4 in serum to confirm the presence of hypothyroidism after a previous screening test or to diagnose for the first time a thyroid alteration. 34.5% of patients (n = 39) were diagnosed at neonatal age within the first month of life, while 41.6% (n = 47) of the patients were diagnosed later in life. The mean age for diagnosis was 15.1 years (range 10 months to 60 years). In the remaining patients, no available data on age at diagnosis was reported. 42.5% of patients (n = 48) were positive for CH. The mean and median values of TSH levels in serum were 86.1mU/L and 29.2 mU/L, respectively. In 14.2% of patients (n = 16) with a diagnosis test, no numerical data was specified, only indications of normal or high TSH levels. One patient showed a high level of TSH, the same patient with elevated TSH at the neonatal screening described previously [39]. As neonatal screening tests, there was no clear consensus on the threshold to establish the TSH levels in serum in diagnosis to confirm hypothyroidism for patients with *NKX2-1-*RD. The cut-off value of TSH in serum was set to a maximum of 6.3 mU/L in 14 studies (30.4%). In one study, the threshold was set at 0.6 mU/L [44] and in the other three studies, the threshold values reached a maximum of 13.1 mU/L [45], 15 mU/L [29] or 24 mU/L [46]. Regarding fT4 levels in serum, they were reported in 24.8% of patients (n = 28) from 19 studies (41.3%). The mean value was 11.6 pmol/L and the median was 12.4 pmol/L. The minimum and maximum thresholds to detect altered fT4 values showed variability among studies. T4 levels were reported in 9 patients from 3 studies [5, 42, 47]. The mean and median values were 72.7 nmol/L and 75.9 nmol/L, respectively. Details about diagnosis tests and hormone levels are presented in S5 File.

### Complementary tests for endocrine, neurological and respiratory alterations in patients with *NKX2-1*-RD

Scintigraphy and ultrasonography were the most frequently used techniques to evaluate the etiology (dysgenesis or gland in situ) of hypothyroidism through the size, location, and echostructure of the thyroid gland, along with the diagnostic tests. Thyroid ultrasonography was used in 33 patients (29.2%) and scintigraphy in 25 (22.1%). It was reported that 32 patients (28.3%) had abnormalities in their thyroid gland: 16.8% of patients (n = 19) had hypoplasia, 4.4% (n = 5) had an ectopic thyroid gland, 3.5% (n = 4) had a thyroid gland with altered uptake, three of them with low uptake and one with no uptake in the neck, 1 patient had hemiagenesis, and 1 had athyreosis. The defect in a patient was not specified [29]. A hypoechogenic nodule was observed in another patient [48]. Perchlorate discharge test and a hearing test were carried out in a study, but no hearing loss was detected, and the thyroid was considered normal [49]. Fetus ultrasound, cordocentesis, and fetal blood sampling were included in the research question as interventions for neonatal screening to cover all the possible evidence about detection procedures in patients with *NKX2-1-*RD. No evidence was finally found in this review about these interventions. Details about thyroid pathology are presented in S5 File.

Regarding neurological abnormalities, 23% of patients underwent additional tests such as MRI, SPET, and SPECT [36, 50–52]. The Denver developmental test, the McCarthy cognitive test, and the WAIS-III scale were utilized for psychomotor, cognitive, and intelligence evaluations, respectively [28, 41]. These tests were conducted during the first 2–3 years of life, when the first symptoms of chorea appeared. Tests for respiratory function were reported in 12.3% of patients (cardiac ultrasound, lung tomography, lung biopsies, and respiratory function tests, among others). These tests were commonly conducted from birth when infants displayed neonatal respiratory distress [13, 33, 39, 53–55].

### Quality assessment of the included studies

According to the assessment tools used in our systematic review, 72.7% (n = 32) of case series and case reports had poor methodological quality, 20.5% (n = 9) had medium quality, and 6.8% (n = 3) had good quality. One cohort study had poor methodological quality [56], whereas the second one had good quality [57]. The scores obtained for each item, as well as the total score for each reference, are shown in S6 File.

### Quality assessment of the evidence

The fact that the available evidence came from case series or case reports downgrades the initial quality. Due to the lack of reported data and inadequate study design, it has not been possible to quantitatively estimate the risk of bias, inconsistency, indirectness, imprecision, and publication bias. The wide variability was due to the lack of concordance among studies in the threshold values of hormone levels and in the age at diagnosis of hypothyroidism. The indirectness of the study was considered low since the available evidence answered the initial research questions. Otherwise, the small sample size and the lack of reporting of interest outcomes lead to imprecision in the results.

In terms of publication bias, conducting an objective assessment was challenging due to the limited availability of data from unpublished studies. However, the reported results consistently align in terms of the detection and diagnosis strategies as well as hormone levels across all patients. The bibliographic searches were designed to be sensitive with the aim of retrieving all the related studies to the criteria of the research questions, but there is the possibility that some of them were lost. The overall quality of the evidence analyzed in this study is considered low due to the previously discussed factors.

## Discussion

This is the first comprehensive review of the evidence on endocrine disease screening and diagnostic methods in *NKX2-1*-RD patients. CH was the most common endocrinological manifestation in almost half of the patients, but neonatal screening detected only a small number. Diagnostic procedures confirmed hypothyroidism mostly later in life. TSH screening and diagnostic threshold values varied greatly. There are cases of transient hypothyroidism during pregnancy. Some patients had additional endocrinological alterations, such as anterior pituitary deficiencies.

We examined 113 patients after reviewing 1012 scientific articles. To maximize search sensitivity, these articles were retrieved from a wide variety of databases using field-specific terms. Since the condition is rare and the search was conducted using articles published after 2002, the number of retrieved studies and patients was optimal [19]. The external validity of the study was ensured by the diverse age of the patients, geographic locations, genetic backgrounds, and clinical manifestations. Only patients with genetic confirmation of *NKX2-1*-RD and hypothyroidism were included to ensure internal validity. Trained and impartial reviewers used a rigorous methodology to select, extract, and evaluate studies.

These findings on *NKX2-1* gene abnormalities align with the literature, which indicates that 50–57% of patients may exhibit the complete triad of symptoms. We found neurological, thyroid, and lung affectation in 57.8% of patients. Lung disorders, hypothyroidism, and central nervous system abnormalities affect 54%, 87%, and 93% of *NKX2-1*-RD patients [43, 58]. Several studies advocate for the widespread screening of newborns to detect thyroid abnormalities and, upon confirmation, recommend further investigation into genetic etiologies, including *NKX2-1*-RD [45, 48, 49]. We found that only 21% of patients have reported screening results for hypothyroidism after birth. Thus, later diagnostic tests were the main tests used to evaluate hypothyroidism in these patients. Notably, the accepted threshold for diagnosing thyroid alteration varies across screening centers. In the UK, blood TSH screening thresholds range from 5 to 12 mU/L, while in Spain, the maximum threshold is 10 mU/L. These thresholds depend on factors such as post-partum sampling time and the screening technology used [13].

Hypothyroidism screening in *NKX2-1*-RD patients should encompass not only neonatal and childhood stages but also extend to the gestational period in affected women. Our study identified cases of transient pregnancy-related hypothyroidism [28–30]. A comprehensive evaluation during gestational hypothyroidism diagnosis is vital, considering associated symptoms like motor developmental delay, abnormal movements, asthma, and recurrent respiratory infections, which could indicate underlying *NKX2-1*-RD.

Thyroid ultrasound and scintigraphy are indispensable in the comprehensive evaluation of hypothyroid patients. It is important to note, however, that thyroid ultrasonography has particular significance in *NKX2-1-*RD due to its ability to precisely measure the dimensions of the thyroid gland of the patients presenting reduced thyroid volume with thyroid hypoplasia. As highlighted by Worth et al. 2021 [59], thyroid scintigraphy is also essential, as it helps distinguish between subtypes of CH.

This review underscores the varied endocrine issues in *NKX2-1*-RD patients. Early detection of TSH deficiency is crucial for managing coexisting hormone deficiencies, including hypothyroidism, and other pituitary irregularities such as growth hormone, cortisol, and gonadotrophin deficiencies. Additionally, some patients exhibited slightly low prolactin levels and cystic pituitary structures [26, 31, 35, 36]. A case series of 23 patients with *NKX2-1* deficiency reported the additional presence of hypothalamic symptoms in some of their patients, including temperature and appetite dysregulation and dysrhythmic sleep [3]. These findings highlight the complexity of *NKX2-1*-RD and the need for comprehensive endocrine screenings to ensure early detection and appropriate management of these multiple endocrine dysfunctions. The co-occurrence of the endocrine abnormalities in *NKX2-1*-RD raises important clinical considerations, as specific interventions for each deficiency may be required to optimize patient outcomes. In addition, understanding the full spectrum of endocrine disruptions associated with *NKX2-1*-RD is essential for developing individualized and effective therapeutic approaches, thereby enhancing the long-term health and well-being of those affected.

The sporadic presence of additional endocrine abnormalities may stem from the variable penetrance of the heterozygous defect within different tissues expressing *NKX2-1*. However, it is noteworthy that the phenotypical expression appears to be fully realized in the singular homozygous patient identified with CH [25]. Furthermore, it is plausible that unidentified mechanisms, such as associated defects, also play a contributory role in this complex phenotypic manifestation. These observations underscore the imperative for a more profound understanding of the underlying mechanisms governing *NKX2-1* regulation and its interactions.

The study presented certain limitations that need to be acknowledged. The low neonatal screening rate in this study could be attributed to variations in screening procedures across countries despite the implementation of NBS programs [60, 61]. This situation means that not all newborns can benefit from early detection. Moreover, a percentage of newborns worldwide are not yet born in a location with a NBS program. In this review, there is a positive trend in adult patients over 40 years of age who did not have neonatal screening, probably because the NBS was not yet established in their country. Younger patients likely underwent screening after birth, but unreported data hinder a clear conclusion about the number detected at birth. Moreover, the absence of standardized thresholds contributes to discordance in positive results for congenital hypothyroidism in newborns [12]. Therefore, negative screening for CH may be overrepresented, and this situation could generate bias in the reporting of data by the authors. The lack of information in neonatal screening is manifest in the retrieved evidence collected for this review. Furthermore, many newborns with CH exhibit minimal or no clinical symptoms at birth. Symptoms such as lethargy, hypotonia, and bradycardia may develop gradually within six weeks, making detection challenging. Clinicians, especially those in regions without newborn screening programs, must remain vigilant for infants displaying suspicious clinical signs, especially if they test positive for *NKX2-1* gene pathogenic variants.

In conclusion, this systematic review provides insights into the detection and diagnosis of endocrine alterations in patients with *NKX2-1*-RD, highlighting the rarity and complexity of the condition. Neonatal screening using blood spots can screen for CH in a few patients (only 20% of patients), but there is a lack of consensus on normal hormone ranges for treating hypothyroidism [16]. Diagnostic tests like MRI, SPET, SPECT, and cognitive evaluations are used to assess neurological and respiratory abnormalities, but limited studies and variability in reporting make quantitative synthesis and overall quality assessment difficult. The inadequate reporting of crucial data pertaining to the detection and diagnosis procedures for hypothyroidism in patients with *NKX2-1*-RD hampers the ability to synthesize the results effectively. Additionally, not all patients had screening for other endocrinological abnormalities documented. Specifically, important information, such as the age at which the tests were conducted and the specific levels of thyroid hormone detected, was not consistently provided across the included studies. Additional limitations of this study encompass the relatively small sample size, the absence of follow-up, and the heterogeneity of the dataset concerning patient demographics, genetic variants, detection methods, and diagnostic criteria employed. Further research and standardization of diagnostic approaches are needed for this rare genetic disorder. This will allow early treatments and reduce clinical symptoms, improving the quality of life of patients.

### Keys for screening and diagnosis of thyroid disorders in patients with *NKX2-1*-RD

1. **Standardize the cut-off threshold for TSH in neonatal screening**. Achieving a clear consensus on the TSH cut-off thresholds will facilitate early detection and prompt further evaluation, ensuring timely diagnosis. Further studies are needed to determine whether the current standard TSH level on blood filter paper at neonatal screening should be modified.
2. **Expansion by healthcare providers of the screening and diagnostic practices for hypothyroidism even after the systematic neonatal screening during infancy, childhood, and in women affected by *NKX2-1*-RD to include the gestational period**. A comprehensive assessment should be conducted in pregnant women with transient hypothyroidism, specifically looking for other manifestations that could be indicative of an underlying *NKX2-1*-RD.
3. **Spread endocrine screening beyond thyroid screening in patients with *NKX2-1*-RD.** Healthcare providers should consider including assessments of clinical manifestations for other endocrine deficiencies, allowing the determination of hormonal impairment. In cases of pituitary hormone deficiency, a systematic cerebral MRI should be performed to look after the size and structure of the pituitary gland.
4. **Facilitate genetic testing.** Given the genetic basis of *NKX2-1*-RD, consider incorporating genetic testing, such as *NKX2-1* gene sequencing, WES, or Array CGH, into the diagnostic workup of patients with CH, particularly those with associated respiratory distress and/or neurological impairment and/or pituitary deficiency. Genetic testing can aid in the identification of specific gene variants or deletions associated with the disorder, thereby facilitating an accurate diagnosis and family counseling.
5. **Improve neurological and respiratory assessment.** In patients with *NKX2-1-*RD, conduct additional tests to assess neurological and respiratory abnormalities, such as MRI, SPET, SPECT, psychomotor, cognitive, and intelligence evaluations. Comprehensive evaluations can aid in the early detection and management of these symptoms, improving patient care.

## Supporting information

S1 File PRISMA checklist. Evidence-based items to report the screening and diagnosis of endocrine diseases in patients with *NKX2-1-*RD.
(DOCX)

S2 File Search strategies. Detailed search strategies used in this study to identify relevant articles related to the screening and diagnosis of endocrine diseases in patients with *NKX2-1*-RD.
(DOCX)

S3 File Included and excluded references. List of final included and excluded articles used in the study and the reasons for the exclusion.
(DOCX)

S4 File Basal characteristics of patients. Comprehensive summary of the baseline characteristics of individual patients included in the study, focusing on patient-level data related to the detection and diagnosis of endocrine diseases in *NKX2-1*-RD.
(DOCX)

S5 File Screening and diagnostic tests. Summary of thyroid pathology, hormone levels, and the results of screening and diagnostic tests for individual patients with *NKX2-1*-RD at the patient level. The table provides valuable insights into the specific thyroid-related abnormalities and hormone levels observed in each patient, along with the outcomes of screening and diagnostic procedures performed as part of the study.
(DOCX)

S6 File Quality assessment. The table displays the quality assessment of the included references concerning the detection and diagnosis of endocrine diseases in patients with *NKX2-1*-RD. **Table A.** Quality assessment of the case reports and case series. **Table B.** Quality assessment of the cohort studies.
(DOCX)
