# Supplementary material for: Systematic review of thyroid function in NKX2-1-related disorders: Screening and diagnosis
Source: PLoS One. 2024 Jul 11;19(7):e0303880. doi: 10.1371/journal.pone.0303880 (PMC11238965; doi:10.1371/journal.pone.0303880)
Supplement: S2 File — Detailed search strategies used in this study to identify relevant articles related to the screening and diagnosis of endocrine diseases in patients with NKX2-1-RD. (DOCX) [file pone.0303880.s002.docx]

**S2. Search strategies.**

**MEDLINE (OVID)**

1. *What are the best procedures for initial screening of endocrine diseases in patients with NKX2-1-related disorders?.*

***Date****: 28/06/23*

***ADVANCE SEARCH*** [***https://www.wolterskluwer.com/en/solutions/ovid/ovid-medline-901***](https://www.wolterskluwer.com/en/solutions/ovid/ovid-medline-901)

| # ▲ | **Searches** |
| --- | --- |
| 1 | exp Thyroid Nuclear Factor 1/ or 'thyroid nuclear factor 1*'.ti,ab,kw or 'thyroid transcription factor*'.ti,ab,kw or 'thyroid-specific enhancer-binding protein*'.ti,ab,kw or 't-ebp'.ti,ab,kw or 'tebp'.ti,ab,kw or 'titf1'.ti,ab,kw or 'titf 1'.ti,ab,kw or 'titf-1'.ti,ab,kw or 'ttf1'.ti,ab,kw or 'ttf 1'.ti,ab,kw or 'ttf-1'.ti,ab,kw |
| 2 | ('nkx2 1*' or 'nkx2-1*' or 'nkx2?1*' or 'nkx 2 1*' or 'nkx 2-1*' or 'nkx 2?1*' or 'nk2 homeobox 1' or 'nkx2 homeodomain transcription*' or 'nk-2 homolog a*' or 'nkx2a*').ti,ab,kw |
| 3 | PAX9 Transcription Factor/ or 'pax9*'.ti,ab,kw or 'pax-9*'.ti,ab,kw or 'pax 9*'.ti,ab,kw or 'pair box 9*'.ti,ab,kw or 14q12*.ti,ab,kw or 14q13*.ti,ab,kw |
| 4 | ((hereditar* adj3 chorea*) or (benign* adj3 chorea*) or 'brain lung thyroid*' or 'brain-lung-thyroid' or 'brain-thyroid-lung' or 'brain thyroid lung').ti,ab,kw |
| 5 | 1 or 2 or 3 or 4 |
| 6 | exp Congenital Hypothyroidism/ or hypothyroidism*.ti,ab,kw |
| 7 | Thyroid Diseases/ge or exp Thyroid Dysgenesis/ge or (thyroid dysgenesis or dyshormonogenesis*).ti,ab,kw |
| 8 | exp Abnormalities, Multiple/ or pedigree.ti,ab,kw or familial.ti,ab,kw or linkage.ti,ab,kw or kindred.ti,ab,kw |
| 9 | 6 or 7 or 8 |
| 10 | 5 and 9 |
| 11 | exp Prenatal Diagnosis/ or 'genetic testing'.ti,ab,kw or 'genotype-phenotyp*'.ti,ab,kw or phenotyp*.ti,ab,kw or haploinsufficienc*.ti,ab,kw or detec*.ti,ab,kw or predic*.ti,ab,kw or early.ti,ab,kw or embry*.ti,ab,kw or fetal.ti,ab,kw or fetus.ti,ab,kw or antenatal.ti,ab,kw or prenatal.ti,ab,kw or perinatal.ti,ab,kw or preterm.ti,ab,kw |
| 12 | exp Cordocentesis/ or Cordocentesis.ti,ab,kw or cord blood.ti,ab,kw |
| 13 | exp Ultrasonography/ or ultrasound.ti,ab,kw or ultrasonog*.ti,ab,kw or us.ti,ab,kw or sonograp*.ti,ab,kw or examination.ti,ab,kw |
| 14 | exp Fetal Blood/ or 'fetal blood'.ti,ab,kw or 'fetal plasma'.ti,ab,kw or spot*.ti,ab,kw |
| 15 | exp Neonatal Screening/ or newborn*.ti,ab,kw or neonat*.ti,ab,kw or birth.ti,ab,kw or infant*.ti,ab,kw |
| 16 | exp Hearing tests/ |
| 17 | 11 or 12 or 13 or 14 or 15 or 16 |
| **18** | **10 and 17** |

1. *What are the best procedures for diagnosis of endocrine diseases in patients with NKX2-1-related disorders?.*

***Date****: 29/06/2023*

***ADVANCE SEARCH*** [***https://www.wolterskluwer.com/en/solutions/ovid/ovid-medline-901***](https://www.wolterskluwer.com/en/solutions/ovid/ovid-medline-901)

| **# ▲** | **Searches** |
| --- | --- |
| 1 | exp Thyroid Nuclear Factor 1/ or 'thyroid nuclear factor 1*'.ti,ab,kw or 'thyroid transcription factor*'.ti,ab,kw or 'thyroid-specific enhancer-binding protein*'.ti,ab,kw or 't-ebp'.ti,ab,kw or 'tebp'.ti,ab,kw or 'titf1'.ti,ab,kw or 'titf 1'.ti,ab,kw or 'titf-1'.ti,ab,kw or 'ttf1'.ti,ab,kw or 'ttf 1'.ti,ab,kw or 'ttf-1'.ti,ab,kw |
| 2 | ('nkx2 1*' or 'nkx2-1*' or 'nkx2?1*' or 'nkx 2 1*' or 'nkx 2-1*' or 'nkx 2?1*' or 'nk2 homeobox 1' or 'nkx2 homeodomain transcription*' or 'nk-2 homolog a*' or 'nkx2a*').ti,ab,kw |
| 3 | PAX9 Transcription Factor/ or 'pax9*'.ti,ab,kw or 'pax-9*'.ti,ab,kw or 'pax 9*'.ti,ab,kw or 'pair box 9*'.ti,ab,kw or 14q12*.ti,ab,kw or 14q13*.ti,ab,kw |
| 4 | ((hereditar* adj3 chorea*) or (benign* adj3 chorea*) or 'brain lung thyroid*' or 'brain-lung-thyroid' or 'brain-thyroid-lung' or 'brain thyroid lung').ti,ab,kw |
| 5 | 1 or 2 or 3 or 4 |
| 6 | exp Congenital Hypothyroidism/ or hypothyroidism*.ti,ab,kw |
| 7 | Thyroid Diseases/ge or exp Thyroid Dysgenesis/ge or (thyroid dysgenesis or dyshormonogenesis*).ti,ab,kw |
| 8 | exp Abnormalities, Multiple/ or pedigree.ti,ab,kw or familial.ti,ab,kw or linkage.ti,ab,kw or kindred.ti,ab,kw |
| 9 | 6 or 7 or 8 |
| 10 | 5 and 9 |
| 11 | exp imaging diagnost/ or imag*.ti,ab,kw or scintigraphy.ti,ab,kw or ultrasonograp*.ti,ab,kw or ultrasound*.ti,ab,kw or sonograp*.ti,ab,kw or scanning.ti,ab,kw or volume*.ti,ab,kw or size*.ti,ab,kw |
| 12 | ((thyrotropin* or 'thyroid hormone*' or th or thyrotropin or tsh or 'thyroid stimulating hormone' or 'free thyroxine' or ft4) adj5 (test* or level* or concentration or measur* or threshold* or analys*)).ti,ab,kw |
| 13 | 11 or 12 |
| **14** | **10 and 13** |
