# Supplementary material for: Systematic review of thyroid function in NKX2-1-related disorders: Screening and diagnosis
Source: PLoS One. 2024 Jul 11;19(7):e0303880. doi: 10.1371/journal.pone.0303880 (PMC11238965; doi:10.1371/journal.pone.0303880)
Supplement: S3 File — List of final included and excluded articles used in the study and the reasons for the exclusion. (DOCX) [file pone.0303880.s003.docx]

**S3. List of the included and excluded studies and reasons for exclusion.**

| **INCLUDED STUDIES** |
| --- |
| 1. Balicza P, Grosz Z, Molnár V, Illés A, Csabán D, Gézsi A, et al. NKX2-1 New Mutation Associated With Myoclonus, Dystonia, and Pituitary Involvement. Front Genet. 2018;9:335. |
| 1. Barnett CP, Mencel JJ, Gecz J, Waters W, Kirwin SM, Vinette KM, et al. Choreoathetosis, congenital hypothyroidism and neonatal respiratory distress syndrome with intact NKX2-1. Am J Med Genet A. 2012;158a(12):3168-73. |
| 1. Barreiro J, Alonso-Fernández JR, Castro-Feijoo L, Colón C, Cabanas P, Heredia C, et al. Congenital hypothyroidism with neurological and respiratory alterations: a case detected using a variable diagnostic threshold for TSH. J Clin Res Pediatr Endocrinol. 2011;3(4):208-11. |
| 1. Carré A, Szinnai G, Castanet M, Sura-Trueba S, Tron E, Broutin-L'Hermite I, et al. Five new TTF1/NKX2.1 mutations in brain-lung-thyroid syndrome: rescue by PAX8 synergism in one case. Hum Mol Genet. 2009;18(12):2266-76. |
| 1. Delestrain C, Aissat A, Nattes E, Gibertini I, Lacroze V, Simon S, et al. Deciphering an isolated lung phenotype of NKX2-1 frameshift pathogenic variant. Front Pediatr. 2022;10:978598. |
| 1. de Filippis T, Marelli F, Vigone MC, Di Frenna M, Weber G, Persani L. Novel NKX2-1 Frameshift Mutations in Patients with Atypical Phenotypes of the Brain-Lung-Thyroid Syndrome. Eur Thyroid J. 2014;3(4):227-33. |
| 1. Doyle DA, Gonzalez I, Thomas B, Scavina M. Autosomal dominant transmission of congenital hypothyroidism, neonatal respiratory distress, and ataxia caused by a mutation of NKX2-1. J Pediatr. 2004;145(2):190-3. |
| 1. Ferrara AM, De Michele G, Salvatore E, Di Maio L, Zampella E, Capuano S, et al. A novel NKX2.1 mutation in a family with hypothyroidism and benign hereditary chorea. Thyroid. 2008;18(9):1005-9. |
| 1. Gentile M, De Mattia D, Pansini A, Schettini F, Buonadonna AL, Capozza M, et al. 14q13 distal microdeletion encompassing NKX2-1 and PAX9: Patient report and refinement of the associated phenotype. Am J Med Genet A. 2016;170(7):1884-8. |
| 1. Gillett ES, Deutsch GH, Bamshad MJ, McAdams RM, Mann PC. Novel NKX2.1 mutation associated with hypothyroidism and lethal respiratory failure in a full-term neonate. J Perinatol. 2013;33(2):157-60. |
| 1. Gras D, Jonard L, Roze E, Chantot-Bastaraud S, Koht J, Motte J, et al. Benign hereditary chorea: phenotype, prognosis, therapeutic outcome and long term follow-up in a large series with new mutations in the TITF1/NKX2-1 gene. J Neurol Neurosurg Psychiatry. 2012;83(10):956-62. |
| 1. Hayasaka I, Cho K, Akimoto T, Ikeda M, Uzuki Y, Yamada M, et al. Genetic basis for childhood interstitial lung disease among Japanese infants and children. Pediatr Res. 2018;83(2):477-83. |
| 1. Hayashi S, Yagi M, Morisaki I, Inazawa J. Identical deletion at 14q13.3 including PAX9 and NKX2-1 in siblings from mosaicism of unaffected parent. J Hum Genet. 2015;60(4):203-6. |
| 1. Hermanns P, Kumorowicz-Czoch M, Grasberger H, Refetoff S, Pohlenz J. Novel Mutations in the NKX2.1 gene and the PAX8 gene in a Boy with Brain-Lung-Thyroid Syndrome. Exp Clin Endocrinol Diabetes. 2018;126(2):85-90. |
| 1. Kharbanda M, Hermanns P, Jones J, Pohlenz J, Horrocks I, Donaldson M. A further case of brain-lung-thyroid syndrome with deletion proximal to NKX2-1. Eur J Med Genet. 2017;60(5):257-60. |
| 1. Kleinlein B, Griese M, Liebisch G, Krude H, Lohse P, Aslanidis C, et al. Fatal neonatal respiratory failure in an infant with congenital hypothyroidism due to haploinsufficiency of the NKX2-1 gene: alteration of pulmonary surfactant homeostasis. Arch Dis Child Fetal Neonatal Ed. 2011;96(6):F453-6. |
| 1. Koht J, Løstegaard SO, Wedding I, Vidailhet M, Louha M, Tallaksen CM. Benign hereditary chorea, not only chorea: a family case presentation. Cerebellum Ataxias. 2016;3:3. |
| 1. Krude H, Schütz B, Biebermann H, von Moers A, Schnabel D, Neitzel H, et al. Choreoathetosis, hypothyroidism, and pulmonary alterations due to human NKX2-1 haploinsufficiency. J Clin Invest. 2002;109(4):475-80. |
| 1. Li M, Li Z, Chen M, Hu Z, Zhou M, Wu L, et al. Novel Missense Variants in PAX8 and NKX2-1 Cause Congenital Hypothyroidism. Int J Mol Sci. 2023;24(1). |
| 1. Lynn MM, Simon D, Kasi AS. Hypoxaemia and interstitial lung disease in an infant with hypothyroidism and hypotonia. BMJ Case Rep. 2020;13(12). |
| 1. Magrinelli F, Rocca C, Simone R, Zenezini Chiozzi R, Jaunmuktane Z, Mencacci NE, et al. Detection and Characterization of a De Novo Alu Retrotransposition Event Causing NKX2-1-Related Disorder. Mov Disord. 2023;38(2):347-53. |
| 1. Makretskaya N, Bezlepkina O, Kolodkina A, Kiyaev A, Vasilyev EV, Petrov V, et al. High frequency of mutations in 'dyshormonogenesis genes' in severe congenital hypothyroidism. PLoS One. 2018;13(9):e0204323. |
| 1. Maquet E, Costagliola S, Parma J, Christophe-Hobertus C, Oligny LL, Fournet JC, et al. Lethal respiratory failure and mild primary hypothyroidism in a term girl with a de novo heterozygous mutation in the TITF1/NKX2.1 gene. J Clin Endocrinol Metab. 2009;94(1):197-203. |
| 1. Monti S, Nicoletti A, Cantasano A, Krude H, Cassio A. NKX2.1-Related Disorders: a novel mutation with mild clinical presentation. Ital J Pediatr. 2015;41:45. |
| 1. Moya CM, Perez de Nanclares G, Castaño L, Potau N, Bilbao JR, Carrascosa A, et al. Functional study of a novel single deletion in the TITF1/NKX2.1 homeobox gene that produces congenital hypothyroidism and benign chorea but not pulmonary distress. J Clin Endocrinol Metab. 2006;91(5):1832-41. 2. Moya CM, Zaballos MA, Garzón L, Luna C, Simón R, Yaffe MB, et al. TAZ/WWTR1 Mediates the Pulmonary Effects of NKX2-1 Mutations in Brain-Lung-Thyroid Syndrome. J Clin Endocrinol Metab. 2018;103(3):839-52. |
| 1. Nakamura K, Sekijima Y, Nagamatsu K, Yoshida K, Ikeda S. A novel nonsense mutation in the TITF-1 gene in a Japanese family with benign hereditary chorea. J Neurol Sci. 2012;313(1-2):189-92. |
| 1. Narumi S, Muroya K, Asakura Y, Adachi M, Hasegawa T. Transcription factor mutations and congenital hypothyroidism: systematic genetic screening of a population-based cohort of Japanese patients. J Clin Endocrinol Metab. 2010;95(4):1981-5. |
| 1. Nattes E, Lejeune S, Carsin A, Borie R, Gibertini I, Balinotti J, et al. Heterogeneity of lung disease associated with NK2 homeobox 1 mutations. Respir Med. 2017;129:16-23. |
| 1. Parnes M, Bashir H, Jankovic J. Is Benign Hereditary Chorea Really Benign? Brain-Lung-Thyroid Syndrome Caused by NKX2-1 Mutations. Mov Disord Clin Pract. 2019;6(1):34-9. |
| 1. Peall KJ, Lumsden D, Kneen R, Madhu R, Peake D, Gibbon F, et al. Benign hereditary chorea related to NKX2.1: expansion of the genotypic and phenotypic spectrum. Dev Med Child Neurol. 2014;56(7):642-8. |
| 1. Prasad R, Nicholas AK, Schoenmakers N, Barton J. Haploinsufficiency of NKX2-1 in Brain-Lung-Thyroid Syndrome with Additional Multiple Pituitary Dysfunction. Horm Res Paediatr. 2019;92(5):340-4. |
| 1. Salerno T, Peca D, Menchini L, Schiavino A, Petreschi F, Occasi F, et al. Respiratory insufficiency in a newborn with congenital hypothyroidism due to a new mutation of TTF-1/NKX2.1 gene. Pediatr Pulmonol. 2014;49(3):E42-4. |
| 1. Salvado M, Boronat-Guerrero S, Hernández-Vara J, Álvarez-Sabin J. [Chorea due to TITF1/NKX2-1 mutation: phenotypical description and therapeutic response in a family]. Rev Neurol. 2013;56(10):515-20. |
| 1. Salvatore E, Di Maio L, Filla A, Ferrara AM, Rinaldi C, Saccà F, et al. Benign hereditary chorea: clinical and neuroimaging features in an Italian family. Mov Disord. 2010;25(10):1491-6. |
| 1. Santos-Silva R, Rosário M, Grangeia A, Costa C, Castro-Correia C, Alonso I, et al. Genetic analyses in a cohort of Portuguese pediatric patients with congenital hypothyroidism. J Pediatr Endocrinol Metab. 2019;32(11):1265-73. |
| 1. Shiohama T, Ohashi H, Shimizu K, Fujii K, Oba D, Takatani T, et al. l-Thyroxine-responsive drop attacks in childhood benign hereditary chorea: A case report. Brain Dev. 2018;40(4):353-6. |
| 1. Tanaka T, Aoyama K, Suzuki A, Saitoh S, Mizuno H. Clinical and genetic investigation of 136 Japanese patients with congenital hypothyroidism. J Pediatr Endocrinol Metab. 2020;33(6):691-701. |
| 1. Tozawa T, Yokochi K, Kono S, Konishi T, Yamamoto T, Nishimura A, et al. A Video Report of Brain-Lung-Thyroid Syndrome in a Japanese Female With a Novel Frameshift Mutation of the NKX2-1 Gene. Child Neurol Open. 2016;3:2329048x16665012. |
| 1. Trevisani V, Predieri B, Madeo SF, Fusco C, Garavelli L, Caraffi S, et al. Growth hormone deficiency in a child with benign hereditary chorea caused by a de novo mutation of the TITF1/NKX2-1 gene. J Pediatr Endocrinol Metab. 2022;35(3):411-5. |
| 1. Uematsu M, Haginoya K, Kikuchi A, Nakayama T, Kakisaka Y, Numata Y, et al. Hypoperfusion in caudate nuclei in patients with brain-lung-thyroid syndrome. J Neurol Sci. 2012;315(1-2):77-81. |
| 1. Veneziano L, Parkinson MH, Mantuano E, Frontali M, Bhatia KP, Giunti P. A novel de novo mutation of the TITF1/NKX2-1 gene causing ataxia, benign hereditary chorea, hypothyroidism and a pituitary mass in a UK family and review of the literature. Cerebellum. 2014;13(5):588-95. |
| 1. Villafuerte B, Natera-de-Benito D, González A, Mori MA, Palomares M, Nevado J, et al. The Brain-Lung-Thyroid syndrome (BLTS): A novel deletion in chromosome 14q13.2-q21.1 expands the phenotype to humoral immunodeficiency. Eur J Med Genet. 2018;61(7):393-8. |
| 1. Villamil-Osorio M, Yunis LK, Quintero L, Restrepo-Gualteros S, Yunis JJ, Jaramillo L, et al. [Brain-lung-thyroid syndrome in a newborn with deletion 14q12-q21.1]. Andes Pediatr. 2021;92(6):930-6. |
| 1. Williamson S, Kirkpatrick M, Greene S, Goudie D. A novel mutation of NKX2-1 affecting 2 generations with hypothyroidism and choreoathetosis: part of the spectrum of brain-thyroid-lung syndrome. J Child Neurol. 2014;29(5):666-9. |
| 1. Zou M, Alzahrani AS, Al-Odaib A, Alqahtani MA, Babiker O, Al-Rijjal RA, et al. Molecular Analysis of Congenital Hypothyroidism in Saudi Arabia: SLC26A7 Mutation Is a Novel Defect in Thyroid Dyshormonogenesis. J Clin Endocrinol Metab. 2018;103(5):1889-98. |

**EXCLUDED STUDIES**

| **Wrong patient population** |
| --- |
| 1. Invernizzi F, Zorzi G, Legati A, Coppola G, D'Adamo P, Nardocci N, Garavaglia B, Ghezzi D. Benign hereditary chorea and deletions outside NKX2-1: What's the role of MBIP? Eur J Med Genet. 2018 Oct;61(10):581-584. 2. Konishi T, Kono S, Fujimoto M, Terada T, Matsushita K, Ouchi Y, Miyajima H. Benign hereditary chorea: dopaminergic brain imaging in patients with a novel intronic NKX2.1 gene mutation. J Neurol. 2013 Jan;260(1):207-13. 3. Kumorowicz-Czoch M, Madetko-Talowska A, Tylek-Lemanska D, Pietrzyk JJ, Starzyk J. Identification of deletions in children with congenital hypothyroidism and thyroid dysgenesis with the use of multiplex ligation-dependent probe amplification. J Pediatr Endocrinol Metab. 2015 Jan;28(1-2):171-6. 4. Li L, Li X, Wang X, Han M, Zhao D, Wang F, Liu S. Mutation screening of eight genes and comparison of the clinical data in a Chinese cohort with congenital hypothyroidism. Endocrine. 2023 Jan;79(1):125-134. 5. Molina MF, Papendieck P, Sobrero G, Balbi VA, Belforte FS, Martínez EB, Adrover E, Olcese MC, Chiesa A, Miras MB, González VG, Pio MG, González-Sarmiento R, Targovnik HM, Rivolta CM. Mutational screening of the TPO and DUOX2 genes in Argentinian children with congenital hypothyroidism due to thyroid dyshormonogenesis. Endocrine. 2022 Jun;77(1):86-101. 6. Ye L, Yin Y, Chen M, Gong N, Peng Y, Liu H, Miao J. Combined genetic screening and traditional newborn screening to improve the screening efficiency of congenital hypothyroidism. Front Pediatr. 2023 May 12;11:1185802. 7. Yu B, Long W, Yang Y, Wang Y, Jiang L, Cai Z, Wang H. Newborn Screening and Molecular Profile of Congenital Hypothyroidism in a Chinese Population. Front Genet. 2018 Oct 29;9:509. |
| **Wrong outcomes** |
| 1. Al Taji E, Biebermann H, Límanová Z, Hníková O, Zikmund J, Dame C, Grüters A, Lebl J, Krude H. Screening for mutations in transcription factors in a Czech cohort of 170 patients with congenital and early-onset hypothyroidism: identification of a novel PAX8 mutation in dominantly inherited early-onset non-autoimmune hypothyroidism. Eur J Endocrinol. 2007 May;156(5):521-9. 2. Costa MC, Costa C, Silva AP, Evangelista P, Santos L, Ferro A, Sequeiros J, Maciel P. Nonsense mutation in TITF1 in a Portuguese family with benign hereditary chorea. Neurogenetics. 2005 Dec;6(4):209-15 3. Hamvas A, Deterding RR, Wert SE, White FV, Dishop MK, Alfano DN, Halbower AC, Planer B, Stephan MJ, Uchida DA, Williames LD, Rosenfeld JA, Lebel RR, Young LR, Cole FS, Nogee LM. Heterogeneous pulmonary phenotypes associated with mutations in the thyroid transcription factor gene NKX2-1. Chest. 2013 Sep;144(3):794-804. 4. Li L, Jia C, Li X, Wang F, Wang Y, Chen Y, Liu S, Zhao D. Molecular and clinical characteristics of congenital hypothyroidism in a large cohort study based on comprehensive thyroid transcription factor mutation screening in Henan. Clin Chim Acta. 2021 Jul;518:162-169. 5. Pérez-Póveda JC, Palacio LG, Arcos-Burgos M. [Description of an endogamous, multigenerational and extensive family with benign hereditary chorea from the Paisa community]. Rev Neurol. 2005 Jul 16-31;41(2):95-8. 6. Provenzano C, Veneziano L, Appleton R, Frontali M, Civitareale D. Functional characterization of a novel mutation in TITF-1 in a patient with benign hereditary chorea. J Neurol Sci. 2008 Jan 15;264(1-2):56-62. |
| 1. Sun F, Zhang JX, Yang CY, Gao GQ, Zhu WB, Han B, Zhang LL, Wan YY, Ye XP, Ma YR, Zhang MM, Yang L, Zhang QY, Liu W, Guo CC, Chen G, Zhao SX, Song KY, Song HD. The genetic characteristics of congenital hypothyroidism in China by comprehensive screening of 21 candidate genes. Eur J Endocrinol. 2018 Jun;178(6):623-633. 2. Thust S, Veneziano L, Parkinson MH, Bhatia KP, Mantuano E, Gonzalez-Robles C, Davagnanam I, Giunti P. Altered pituitary morphology as a sign of benign hereditary chorea caused by TITF1/NKX2.1 mutations. Neurogenetics. 2022 Apr;23(2):91-102. 3. Wang F, Liu C, Jia X, Liu X, Xu Y, Yan S, Jia X, Huang Z, Liu S, Gu M. Next-generation sequencing of NKX2.1, FOXE1, PAX8, NKX2.5, and TSHR in 100 Chinese patients with congenital hypothyroidism and athyreosis. Clin Chim Acta. 2017 Jul;470:36-41. 4. Young LR, Deutsch GH, Bokulic RE, Brody AS, Nogee LM. A mutation in TTF1/NKX2.1 is associated with familial neuroendocrine cell hyperplasia of infancy. Chest. 2013 Oct;144(4):1199-1206. |

| **Wrong study design** |
| --- |
| 1. Nettore IC, Cacace V, De Fusco C, Colao A, Macchia PE. The molecular causes of thyroid dysgenesis: a systematic review. J Endocrinol Invest. 2013 Sep;36(8):654-64. |

**Wrong publication type**

| 1. Akkari M, Ben Rhouma H, Klaa H, Rouissi A, Kraoua I, Turki I. Benign hereditary chorea: A Tunisian family case presentation. Mov. Disord. 2019;34 (Tunis, Tunisia):S123. 2. Auyeung V, Planer BC, Chartoff A, Oundjian N. The link between respiratory failure and congenital hypothyroidism. Center for Children, Joseph M. Sanzari Children's Hospital, Hackensack University Medical Center, Hackensack, NJ, United States): Thyroid. 2010. A52-A53. 3. Baldan F, Cavaliere E, Gortan AJ, Passon N, Fabbro D, Marin D, Carecchio M, Credendino SC, Gallo R, Cogo P, Damante G, De Vita G. A case of familial brain-lung-thyroid syndrome due to a NKX2.1 run-on mutation. European Journal of Human Genetics 2022;30(SUPPL 1):150. 4. Barbian ME, Piazza A, Gauthier T, Williams H. Use of genetic sequencing in premature infant with persistent respiratory failure. J. Invest. Med. 2018;66(2):51. 5. Bhatia P, Coffman K. Autosomal dominant familial chorea arising from a novel deletion of chromosome 14q13.3. Ann. Neurol. 2012;72. Pittsburgh, PA, United States):S184. 6. Burglen L, Ravelli C, Louha M, Qebibo L, Afenjar A, Mignot C, Rodriguez D, Doummar D. Childhood onset chorea: an overview of genetic etiologies in a series of 85 patients. European Journal of Human Genetics 2023;31():197. 7. Burke A, Mantuano E, Bhatia KP, Veneziano L, Giunti P. Novel de novo mutation causing benign hereditary chorea with hypothryoidism and a pituitary mass. J. Neurol. Neurosurg. Psychiatry 2012;83(Insititute of Neurology, United Kingdom):A11. 8. Coon Elizabeth A, Ahlskog JE, Patterson MC, Niu Z, Milone M. Expanding phenotypic spectrum of NKX2-1–related disorders—Mitochondrial and immunologic dysfunction. JAMA Neurology 2016;73(2):237-238. 9. Deshpande A, Dutta S, Singha A, Mukhopadhyay P, Ghosh S. Abstract 125: Congenital hypothyroidism: Clinical profile and genetic abnormalities. Indian Journal of Endocrinology and Metabolism. 2022. 26(Suppl 8):p S53-S54. 10. Deterding RR, Dishop M, Uchida DA, Stephan M, Williames L, Lebel RR, Halbower AC, Rosenfeld J, Moffitt D, Wert SE, Nogee L. Thyroid transcription factor 1 gene abnormalities: An under recognized cause of children's interstitial lung disease. Am. J. Respir. Crit. Care Med. 2010;181(1). 11. Doummar D, Chantot Bastauraud S, Heron B, Lion Francois L, Devos D, Gras D, Echenne B, Billette De Villemeur T, Vidailhet M. Benign hereditary chorea: Report of three cases illustrating the contribution of array CGH for molecular diagnosis. Dev. Med. Child Neurol. 2012;54 (Neuropediatrie, Hôpital Trousseau, Paris, France):40. 12. Du Souich C, Scocchia A, Gall K, Hathaway J, Taylor A, Huusko J, Bernal M, Saarinen I, Schleit J, Paananen J, Myllykangas S, Koskenvuo J. Characterization of molecular diagnostic findings in an unselected cohort with suspected congenital hypothyroidism or resistance to thyroid hormone. European Journal of Human Genetics 2023;31():120. 13. El Taoum K, Com G, Warren, R. An infant with congenital hypothyroidism and labored breathing: A case report. Am. J. Respir. Crit. Care Med. 2014;189 (University of Arkansas for Medical Sciences, Little Rock, AR, United States). 14. Ferrara, JM, Adam OR, Kirwin SM, Houghton, DJ, Litvan I. Brain-lung-thyroid disease, (BLT) - Clinical features of a kindred with a novel TITF-1 mutation Mov. Disord. 2011;26 (Louisville, KY, United States):S33-S34. 15. Ferreira SH, Pereia S, Jacob S, Abreu M, Gonçalves D, Sampaio M, Leão M, Castro-Correia C, Fontoura, M. Congenital hypothyroidism and Brain-Lung-Thyroid syndrome. Cogent Med. 2017;4(1). 16. Gonzalez A, Villafuerte B, Jimenez P, Arguinzoniz L, De La Luz Ruiz M, Villarroel C, Calzada R, Robles C, Moreno JC. Brain-lung-thyroid syndrome (BLTS) in a mexican patient with a novel intragenic deletion in NKX2-1. Horm. Res. Paediatr. 2017;88 (National Institute of Pediatrics INP, Mexico City, Mexico):592. 17. Graf S, Bösch N, Bachmann S, Zumsteg U, Heinimann K, Szinnai G. Familial brain-lung-thyroid syndrome due to a new NKX2-1 mutation p.Q172L causing disabling benign hereditary chorea. Horm. Res. Paediatr. 2012;78 (University Children's Hospital Basel, Paediatric Endocrinology, Basel, Switzerland):127. 18. Hashemipour M, Hopvsepian S. Genetic studies in congenital hypothyroidism: A regional study. Horm. Res. Paediatr. 2014;82 (Isfahan Endocrine and Metabolism Research Center, Isfahan University of Medical Sciences, Isfahan, Iran):318. 19. Haubenberger D, Bauer P, Lieba-Samal D, Zimprich A, Auff E, Pirker W. A novel NKX2A-mutation causing benign hereditary chorea is associated with non-progressive striatal D2-receptor dysfunction. Mov. Disord. 2012;27 (Vienna, Austria):S327. 20. Hermanns P, Kumorowicz-Czoch M, Pohlenz J. Mutations in the TTF1 and the PAX8 genes in a boy with thyroid dysgenesis, respiratory and neurological disorders. Horm. Res. Paediatr. 2013;80 (Johannes Gutenberg University Medical School, Department of Pediatrics, Mainz, Germany):194. 21. Jung SY, Lee J. Analysis of hypothyroidism NGS test in Korean patients with congenital hypothyroidism in a single center. Horm. Res. Paediatr. 2021;94(SUPPL 1):172-173. 22. Kardelen AD, Işik FB, Özturan EK, Sözügüzel MD, Öztürk AP, Poyrazoǧlu S, Parlayan C, Cangül H, Baş F, Darendeliler F. The investigation of genetic etiology in familial cases with congenital hypothyroidism. Horm. Res. Paediatr. 2019;91(Istanbul University, Istanbul Faculty of Medicine, Pediatric Endocrinology Department, Istanbul, Turkey):199. 23. Lenherr N, Vuissoz JM, Heinimann K, Szinnai G. Brain-lung-thyroid syndrome-update on the clinical spectrum of a heterogeneous disorder. Horm. Res. Paediatr. 2015;84 (Division of Paediatric Endocrinology, University Children's Hospital, Basel, Switzerland):123-124. 24. Levaillant L, Bouhours-Nouet N, Illouz F, Bouzamondo N, Rodien P, Prunier-Mirebeau D, Coutant R. Genetic analyses in patients having congenital hypothyroidism with gland-in-situ by nextgeneration sequencing. Horm. Res. Paediatr. 2021;94(SUPPL 1):61. 25. Le Moine B, Liptzin D, Galambos C, Browne L, Weinman J. The high resolution computed tomography characteristics of thyroid transcription factor 1 deficiency. Pediatr. Radiol. 2017;47 (Children's Hospital Colorado, Aurora, CO, United States):S138. 26. Limon MA, Parveen S, Rehman A, Jakoby M. Abstract #1001910: Brain-Lung-Thyroid Syndrome (BTLS) Diagnosed in an Adult and Caused by a Newly Recognized Pathogenic Variant of Thyroid Transcription Factor-1 (TTF-1). Endocrine Practice 2021;27(6):S157. 27. Löf C, Patyra K, Kuulasmaa T, Kleinau G, Jäschke H, Undeutsch H, Kero A, Krude H, Pursiheimo J, Jääskeläinen J, Toppari J, Ignatius J, Laakso M, Kero J. Detection of novel gene variations in congenital hypothyroidism with targeted next-generation sequencing. Thyroid 2015;25 (Department of Physiology, University of Turku, Turku, Finland):A14. 28. Lourenço L, Reis E Melo A, Gonçalves D, Sampaio M, Correia C, Guardiano M. Coreoathetosis and congenital hypothyroidism. Cogent Med. 2017;4(1). 29. Lumsden DE, Turnbull J, Josifov DJ, Dlamini N, Jungbluth H. Choreoathetoid movement disorder due to a 14q13 deletion including the NKX2-1 gene encoding thyroid transcription factor-1 (TITF1). Dev. Med. Child Neurol. 2012;54 (Paediatric Neurology, Evelina Children's Hospital, London, United Kingdom):16. 30. MacLean J, Luc Q, Ramos-Platt L, Saitta S, Quindipan C. NKX2-1-related disorder with cerebral folate deficiency. Mov. Disord. 2019;34 (Los Angeles, CA, United States):S216. 31. Maristella S, Deborah B, Gabor S, Karl H, Britta S, Maya S, Emma FC. Brain-lung-thyroid syndrome due to a new NKX2-1 mutation. Swiss Med. Wkly 2019;149 (Department of Pediatric Endocrinology, Diabetology and Metabolism, Inselspital, Bern University Children's Hospital, Switzerland):22S. 32. Meijer IA, Michaud J, Tran L, Rossignol E., Van Vliet G, Deladoëy J, Chouinard S, Bernard G. Novel mutations in TITF1 cause benign hereditary chorea, hypothyroidism and neonatal respiratory distress syndrome. Ann. Neurol. 2014;76 (Montreal, QB, Canada):S223-S224. 33. Morandini M, Leger J, Polak M, Castanet M, Congenital H. Psychomotor delay in patients with congenital hypothyroidism: Inadequate treatment or genetic syndrome?. Horm. Res. Paediatr. 2010;74 (Université Paris Descartes, Pediatric Endocrinology Unit, Paris, France):88-89. 34. Nathan N, Borie R, Jovien S, Doummar D, Louha M, Beucher J, Henriat M, Breton E, Clement A. Phenotype heterogeneity in a familial “brain lung thyroid syndrome” related to a novel NKX-2.1 mutation. Eur. Respir. J. 2016;48. 35. Papathoma E, Dasopoulou M, Anatolitou F, Georgiou K, Vizirakis M, Koumenidou M. Acute renal failure, congenital hypothyroidism and pax8 genotype in neonate: A case report. J. Perinat. Med. 2013;41 (Saint Sophia Children's Hospital, Athens, Neonatology Department, Greece). 36. Park J, Kim, S, Lee J. Next Generation Sequencing Analysis of Congenital Hypothyroidism Patients in A Single Tertiary Center. Hormone Research in Paediatrics 2022;95():412. |
| --- |
| 1. Patianna VD, Predieri B, Garavelli L, Fusco C, Madeo SF, Bruzzi P, Iughetti L. A novel mutation in the TITF1 gene in a child with benign hereditary chorea. Horm. Res. Paediatr. 2014;82 (Department of Medical and Surgical Sciences Mother, Children and Adult, University of Modena and Reggio Emilia, Modena, Italy):167. 2. Peake D, Cassidy D, Mckee S, King MD, Kuria MA. A novel TITF1 mutation in benign hereditary chorea. Dev. Med. Child Neurol. 2012;54 (Paediatric Neurology, Royal Belfast Hospital for Sick Children, Belfast, United Kingdom):82. 3. Peall KJ, Lumsden D, Morris HR, Jungbluth H, Kurian MA. NKX2-1 mutations in brain-lung-thyroid syndrome: A case series. Dev. Med. Child Neurol. 2013;55 (Neurology, University Hospital of Wales, Cardiff, United Kingdom). 4. Reem I, Jacqueline S, Bishay Lara C. PULMONARY HYPERTENSION in A LIMPING CHILD: A CASE of URGEN-C. Pulmonary Circulation 2022;12(2). 5. Simões AS, De Brito Chagas J, Dias A, Dionísio MT, Pinto C, Mirante MA, Madureira N, Almeida S, Maia S, Salgado M. Interstitial Lung Disease in a Full-term Neonate Presenting with ARDS and Hypothyroidism: A Case of an NKX 2-1-related Disorder. Klin. Padiatr. 2021;233(5):258-261 6. Shammas C, Neocleous V, Phylactou LA, Gennata ET. A novel NKX2.1 mutation in a family with congenital hypothyroidism (CH). Horm. Res. Paediatr. 2013;80 (Institute of Neurology and Genetics, Molecular Genetics, Function and Therapy, Nicosia, Cyprus):353. 7. Stoeva I, Thorwarth A, Stoilov B, Krude H. NKX2-1 p.Asp266Argfs142X de novo mutation in a girl with congenital hypothyroidism (CH): Phenotypic description. Horm. Res. Paediatr. 2015;84 (University Pediatric Hospital Sofia, Medical University Sofia, Sofia, Bulgaria):556-557. 8. Stoupa A, Gueriouz M, Karyiawasam D, Hanein S, Bole-Feysot C, Deladoey J, Szinnai G, Polak Michel, Carre A.  The Paris-Image “Hypothyseq NGS panel” is now functional and brings valuable information on the genetics in a large cohort of children with congenital hypothyroidism. Hormone research in paediatrics. 2017. 9. Sutedja P, Hung D, Alcausin M, Peters GB. A three generation family with del(14)(q13.3q21.3) with variable phenotypic expresión. Twin Res. Hum. Genet. 2009;12(5):526. 10. Villafuerte B, De Benito DN, Lacamara N, Garcia M, Lumbreras C, De Randamie R, Nevado J, Moreno, JC. Identification of a “Cryptic” de novo deletion in NKX2.1 in the Brain-Lung-Thyroid syndrome using genomic SNP arrays. Horm. Res. Paediatr. 2016;86 (Molecular Laboratory, Institute for Medical and Molecular Genetics (INGEMM), La Paz University Hospital, Madrid, Spain):491. 11. Villafuerte B, Gonzalez A, Moya C, Garzon L, Herranz A, De Benito DN, Gallego M, Palomares M, Nevado J, Moreno JC. NKX2-1 gene defects in a pediatric cohort with suspected brain-lung-thyroid syndrome. Horm. Res. Paediatr. 2017;88 (Thyroid Molecular Laboratory, Institute for Medical and Molecular Genetics (INGEMM), La Paz University Hospital, Madrid, Spain):376-377. 12. Wouters L, De Bruyn G, De Waele , Jansen K, Francois I, Reynaert N, Buyse G, Lagae L, Goemans N. The brain-lung-thyroid syndrome as a rare cause of chorea: Case report of a novel mutation in the thyroid transcription factor-1 (TITF-1) gene. Eur. J. Paediatr. Neurol. 2013;17 (UZ Leuven, Belgium):S104. |
| 1. Yang J, Chung WY, Oh SH, Seo GH, Kim JR, Yu J. A case of permanent congenital hypothyroidism with NKX2-1 mutation and optic nerve thickness. Hormone Research in Paediatrics 2022;95():587. |
| 1. Yilmaz AA, Erdeve SS, Yuksel D, Oztoprak U, Cetinkaya S. A rare cause of congenital hypothyroidism: Brain-lung-thyroid syndrome. Horm. Res. Paediatr. 2021;94(SUPPL 1):413-414. |
| \| **Non-available text** \| \| --- \| \| 1. Heidari MM, Madani Manshadi SA, Eshghi AR, Talebi F, Khatami M, Bragança J, Ordooei M, Chamani R, Ghasemi F. Mutational and bioinformatics analysis of the NKX2.1 gene in a cohort of Iranian pediatric patients with congenital hypothyroidism (CH). Physiol Int. 2022 Jun 7;109(2):261-277. 2. Kojima Y, Atobe M, Aoki Y, Suzuki M, Itomi K, Tanaka T, Saitoh S. A case of brain-lung-thyroid syndrome showing atypical symptoms. No To Hattatsu 2021;53(1):44-48 3. Peters B, Van Mossevelde PWJ, Van Trotsenburg ASP. Neonatal respiratory distress of unknown origin: Do not forget the thyroid gland. Tijdschr. Geneeskd. 2017;73(5):289-294. \|  1. Zhao SX. Comprehensive screening of causative genes in Chinese han patients with congenital hypothyroidism. Eur. Thyroid J. 2019;8(SUPPL 1):79. |
| **Wrong language** |
| 1. Liang R, Ou S, Ding Y, Liu C. A case of brain**-**lung**-**thyroid syndrome. Zhong Nan Da Xue Xue Bao Yi Xue Ban. 2022 Mar 28;47(3):396-400. |
| 1. Wang J, Li H, Yuan S, Sun X, Peng X, Hu Y. [Clinical phenotype and genetic analysis of a child with 14q12q13 microdeletion syndrome manifesting as congenital hypothyroidism]. Zhonghua Yi Xue Yi Chuan Xue Za Zhi. 2023 May 10;40(5):598-603. |
