# Supplementary material for: Systematic review of thyroid function in NKX2-1-related disorders: Screening and diagnosis
Source: PLoS One. 2024 Jul 11;19(7):e0303880. doi: 10.1371/journal.pone.0303880 (PMC11238965; doi:10.1371/journal.pone.0303880)
Supplement: S4 File — Comprehensive summary of the baseline characteristics of individual patients included in the study, focusing on patient-level data related to the detection and diagnosis of endocrine diseases in NKX2-1-RD. (DOCX) [file pone.0303880.s004.docx]

**S4. Summary of basal characteristics. Patient level**

| **Patient and reference** | **Age^a^** | **NKX2-1 mutation (gene test^b^)** | **Inherited^c^** | **Endocrine alterations^d^** | **Neurodevelopmental disorders** | **Respiratory affections** | **Other clinical data** |
| --- | --- | --- | --- | --- | --- | --- | --- |
| **Balicza_2018_PII/2** | Adult | Frameshift, *NKX2-1* (NM_003317):  c.338G>A, p.Trp113*  (sequencing) | NA | H, testosterone and luteinizing hormone deficiency, hypogonadism, pituitary disfunction. | Chorea, ataxia, dyskinesia, hyperkinesia, dystonia, dysarthria, developmental delay. | Asthma. | Hepatic steatosis. |
| **Barnett_2012_P1** | Child | Deletion 14q13.1-q13.3 (CGH array) | D | CH, hypoplastic pituitary gland. | Chorea, ataxia, muscular hypotonia, hyperkinesia. | Neonatal respiratory distress, respiratory infections, chronic cough. | Developmental delay, esotropia, facial dysmorphism. |
| **Barreiro_2011_P1** | Child | Splicing, *NKX2-1* (ENST00000354822.7):c.464-1G>A (NA) | NA | CH | Chorea, mild hypotonia, dystonia, motor retardation. | Neonatal respiratory distress, bronchial hyperactivity, bronchiolitis. | Removal of tonsils and adenoids. |
| **Carré_2009_P1** | Child | Deletion 14q13 (FISH) | D | Mild CH (CpH later) | Chorea, ataxia, muscular hypotonia. | Neonatal respiratory distress, pulmonary infections. | Developmental delay, corpus callosum agenesis. |
| **Carré_2009_P2TA** | Adolescent | Splicing, *NKX2-1* (ENST00000354822.7):c.464-1G>A (sequencing) | D | CH | Chorea, muscular hypotonia. | Neonatal respiratory distress, pulmonary infections. | Developmental delay. |
| **Carré_2009_P3TB** | Adolescent | Splicing, NKX2-1, (NM_?), c.376-2A>G/p.? (sequencing) | D | CH | Chorea. | Neonatal respiratory distress. | NA |
| **Carré_2009_P4** | Adolescent | Missense, *NKX2-1* (NM_003317.4):  c.526C>G/  p.Leu176Val (sequencing) | D | H | Chorea, muscular hypotonia. | Bronchitis. | Developmental delay. |
| **Carré_2009_P5** | Child | Missense, NKX2-1 (NM_003317.4):  c.605C>T/  p.Pro202Leu (sequencing) | D | CH | Chorea, muscular hypotonia. | NA | Developmental delay. |
| **Carré_2009_P6** | NA | Missense, *NKX2-1* (NM_003317.4):  c.629A>C/  p.Gln210Pro (sequencing) | D | CH | Chorea, muscular hypotonia. | NA | Developmental delay. |
| **Delestrain_2023_P2** | NA | Frameshift, *NKX2-1* (NM_003317.4):  c.254dup/  p.Tyr86LeufsTer323 (sequencing) | D | CH | Chorea, hypotonia, motor delay, chorea. | Neonatal respiratory distress, hyaline membrane disease. | NA |
| **de Filippis_2014_P3** | NA | Frameshift, *NKX2-1*(NM_003317.3):c.177dup/p.His60Alafs*349  (sequencing) | D | CH | Chorea, spasticity. | NA | NA |
| **de Filippis_2014_P4** | NA | Frameshift, *NKX2-1*(NM_003317.3): c.153_166del/ p.Glu52SerfsTer382 (sequencing) | D | Idiopathic Mild H. | Chorea, muscular hypotonia. | NA | Neurological deterioration. |
| **Doyle_2004_P I1** | Adult | Missense, *NKX2-1* (NM_001079668.3),c.740A>G/p.Lys247Arg (sequencing) | NA | H | Muscular hypotonia. | Pneumonia. | Stammering. |
| **Doyle_2004_P II2** | NA | Missense, *NKX2-1* (NM_001079668.3),c.740A>G/p.Lys247Arg (sequencing) | M | H | Cerebral palsy. | NA | Stammering, constipation. |
| **Doyle_2004_P III2** | Child | Missense, *NKX2-1* (NM_001079668.3),c.740A>G/p.Lys247Arg (sequencing) | M | CH | Chorea, ataxia, dysarthria. | Neonatal respiratory distress. | Developmental delay. |
| **Doyle_2004_P III3** | Child | Missense, *NKX2-1* (NM_001079668.3),c.740A>G/p.Lys247Arg (sequencing) | M | CH | Chorea, ataxia, dysarthria. | Neonatal respiratory distress. | Developmental delay. |
| **Ferrara_2008_P1 (IV-1) (proband)** | NA | Frameshift, *NKX2-1*(NM_001079668.3), c.524C>A/p.Ser175Ter (sequencing) | M | CH | Motor delay. | Neonatal respiratory distress. | Bilateral pyelectasis, magabladder, foramen ovale, vesicouretheral reflux. |
| **Ferrara_2008_P2 (III-1)** | NA | Frameshift, *NKX2-1*(NM_001079668.3), c.524C>A/p.Ser175Ter (sequencing) | P | H | Chorea. | NA | NA |
| **Ferrara_2008_P3 (II-3)** | NA | Frameshift, *NKX2-1*(NM_001079668.3), c.524C>A/p.Ser175Ter (sequencing) | D | Mild H | Motor delay, hyperkinesia. | NA | Meningitis. |
| **Gentile_2016_P1** | NA | Deletion 14q13.2q21.1 (CGH array) | D | SH | NA | NA | Low fetal growth, nasal obstruction, craniofacial dysmorphisms, recurrent rhinitis, reduction CD3/CD8 ratio, increased CD4/CD8 ratio. |
| **Gillett_2013_P1** | Neonate | Missense, *NKX2-1* (NM_001079668.3), c.711C>G/p.Ile237Met(sequencing) | D | CH | NA | Neonatal respiratory distress, hypotension. | Ventricular dysfunction. The patient passed away at 28 days (pulmonary insufficiency). |
| **Gras_2012_P10_(D)** | Child | Splicing, *NKX2-1* (NM_001079668.3), c.373+1_373+4del/p.? (sequencing) | M | CpH | Chorea, muscular hypotonia. | NA | Developmental delay. |
| **Gras_2012_P11_(D)** | Child | Splicing, *NKX2-1* (NM_001079668.3), c.373+1_373+4del/p.? (sequencing) | M | CpH | Chorea, muscular hypotonia. | Asthma. | Developmental delay. |
| **Gras_2012_P13_(E)** | Adult | Missense, *NKX2-1* (NM_001079668.3), c.671T>G/p.Leu224Arg (sequencing) | M | CpH | Chorea, motor tics, myoclonus. | NA | Learning difficulties |
| **Gras_2012_P15_(E)** | Adult | Missense, *NKX2-1* (NM_001079668.3), c.671T>G/p.Leu224Arg (sequencing) | M | CpH | Chorea, muscular hypotonia, dystonia, tremor. | Asthma. | Learning difficulties |
| **Gras_2012_P18_(E)** | Child | Missense, *NKX2-1* (NM_001079668.3), c.671T>G/p.Leu224Arg (sequencing) | M | CpH | Chorea. | Asthma. | Learning difficulties, ADHD. |
| **Gras_2012_P19_(E)** | Adult | Missense, *NKX2-1* (NM_001079668.3), c.671T>G/p.Leu224Arg (sequencing) | NA | H | Chorea, muscular hypotonia, dystonia. | Asthma. | NA |
| **Gras_2012_P2_(A)** | Adult | Frameshift, *NKX2-1* (NM_001079668.3), c.257dup/p. His86GlnfsTer353 (sequencing) | M | H | Chorea, muscular hypotonia, dystonia, | Asthma. | Learning difficulties. |
| **Gras_2012_P20_(E)** | Adult | Missense, *NKX2-1* (NM_001079668.3), c.671T>G/p.Leu224Arg (sequencing) | NA | H | Chorea, muscular hypotonia, dystonia, tremor. | Asthma. | Learning difficulties. |
| **Gras_2012_P21** | Adolescent | Frameshift, *NKX2-1* (NM_001079668.3),c.399delC/p.Met134Ter (sequencing) | NA | CpH | Chorea, muscular hypotonia. | Asthma. | Learning difficulties. |
| **Gras_2012_P22** | Adolescent | Missense, *NKX2-1* (NM_001079668.3), c.526C>G/p.Leu176Val (sequencing) | NA | H | Chorea, muscular hypotonia, dystonia, myoclonus. | Asthma. | Speech delay, palatal cleft, dental agenesis. |
| **Gras_2012_P23** | Child | Frameshift, *NKX2-1* (NM_001079668.3), c.786_787del/ p.Asp262GlufsTer176 (sequencing) | D | CH | Chorea, muscular hypotonia, myoclonus. | Neonatal respiratory distress. | NA |
| **Gras_2012_P24** | Child | Missense, *NKX2-1* (NM_001079668.3), c.643T>G/p.Tyr215Asp(sequencing) | D | CH | Chorea. | NA | NA |
| **Gras_2012_P26** | Adolescent | Missense, *NKX2-1* (NM_001079668.3), c.719A>C/p.Gln240Pro (sequencing) | NA | CH | Chorea, muscular hypotonia. | NA | NA |
| **Gras_2012_P27** | Adult | Deletion 14q13.2q21.2 (6.2 Mb) (CGH array) | D | H | Chorea, muscular hypotonia. | NA | Learning difficulties, ADHD. |
| **Gras_2012_P28** | Adolescent | Deletion 14q13.3 (0.3 Mb) (CGH array) | D | CpH | Chorea, muscular hypotonia, motor and vocal tics. | NA | Learning difficulties. |
| **Gras_2012_P3_(A)** | Child | Frameshift, *NKX2-1* (NM_001079668.3), c.257dup/ p.His86GlnfsTer353 (sequencing) | NA | H | Chorea, muscular hypotonia. | Lung cancer. | Learning difficulties. |
| **Gras_2012_P4_(B)** | Adolescent | Frameshift, *NKX2-1* (NM_001079668.3), c.732C>A/ p.Tyr244Ter (sequencing) | M | CpH | Chorea, muscular hypotonia. | NA | Learning difficulties, ADHD. |
| **Gras_2012_P7_(B)** | Adult | Frameshift, *NKX2-1* (NM_001079668.3), c.732C>A/ p.Tyr244Ter (sequencing) | NA | CpH | Chorea, myoclonus. | NA | NA |
| **Hayasaka_2018_P7** | Child | Frameshift, *NKX2-1* (NM_001079668.3), c.1117C>T/p.Gln373Ter (sequencing) | NA | H | NA | Pneumonia, cyanosis, ground glass opacification in lung. | NA |
| **Hayasaka_2018_P8** | Child | Frameshift, *NKX2-1* (NM_001079668.3), c.1016_1017insCCATCTCCGTGGGCAGCGG/p.Gly339fs (sequencing) | D | H | NA | Interstitial lung disease, cyanosis, glass opacification in lung. | Developmental delay. |
| **Hayasaka_2018_P9** | Child | Frameshift, *NKX2-1* (NM_001079668.3), c.954_958GCAGG>  CAG/p.Gln318fs (sequencing) | D | H | NA | Interstitial lung disease, pulmonary alveolar proteinosis, anomalous pulmonary venous connection, cyanosis, ground glass opacification in lung. | Developmental delay. |
| **Hayashi_2015_PII1** | NA | Deletion 14q13.3q13.3 (BAC array) | P | H | Chorea, motor delay. | Pulmonary hypertension. | Oligodontia, developmental, speech delay. |
| **Hayashi_2015_PII2 (proband)** | NA | Deletion 14q13.3q13.3 (BAC array) | P | H | Chorea, motor delay. | Pulmonary hypertension. | Oligodontia, developmental speech delay. |
| **Hermanns_2018_P1** | Child | Frameshift, *NKX2-1*(ENST00000522719.4):c.986_987insG  (p.Asp329Glufs) (sequencing) | D | CH | Psychomotor retardation. | Severe respiratory failure. | Epilepsy, cerebral palsy. |
| **Kharbanda_2017_P1** | Child | Deletion  14q13.2q13.3, not involving NKX2-1 (CGH array) | D | CH | Chorea, muscular hypotonia, dystonia-myoclonic movement disorder, hypermobility. | NA | Fetal finger pads, prominent forehead. |
| **Kleinlein_2011_P1** | Child | Frameshift, *NKX2-1* (NM_001079668.3), c.278_308del/ p.Ala93GlyfsTer22 (sequencing) | D | Mild CH | NA | Neonatal respiratory distress, lung ground glass opacity, taquipnea, cardiac defect, vascular resistance. | The patient passed away at of 10 months (respiratory and heart failure). |
| **Koht_2016_P II:4** | NA | Missense, *NKX2-1* (NM_001079668.3), c.671T>G/p.Leu224Arg (NA) | M | H | Chorea, dystonia, hyperkinetic movements, delayed motor skills. | Asthma. | NA |
| **Koht_2016_P II:7** | NA | Missense, *NKX2-1* (NM_001079668.3), c.671T>G/p.Leu224Arg (NA) | M | H | Chorea, dystonia, mild ataxia, hyperkinetic movements, delayed motor skills. | Asthma. | NA |
| **Koht_2016_P III:6 (index)** | Adult | Missense, *NKX2-1* (NM_001079668.3), c.671T>G/p.Leu224Arg (CGH array) | M | CpH | Chorea, mild ataxia, hyperkinetic movements, delayed motor skills, myoclonus. | Asthma. | Low verbal and memory attention. |
| **Koht_2016_P III:7** | NA | Missense, *NKX2-1* (NM_001079668.3), c.671T>G/p.Leu224Arg (NA) | M | CpH | Hyperkinetic movements, delayed motor skills, chorea, dystonia. | Respiratory infections | NA |
| **Koht_2016_P IV:3** | NA | Missense, *NKX2-1* (NM_001079668.3), c.671T>G/p.Leu224Arg (NA) | M | CpH | Chorea, hyperkinetic movements, delayed motor skills. | Asthma. | Low verbal and memory attention. |
| **Krude_2002_P1** | Adolescent | Deletion 14q11.2q13.3 (sequencing) | NA | CH | Chorea. | Neonatal respiratory distress, pulmonary infections. | NA |
| **Krude_2002_P2** | Adolescent | Missense, *NKX2-1*(NM_?) (G2626T)/p.? (sequencing) | D | CH  (euthyroid hyperthyreotropinemia at 15m) | Chorea. | Neonatal respiratory distress, pulmonary infections. | NA |
| **Krude_2002_P3** | Child | Frameshift *NKX2-1*(NM_?) (259insGG)/p.? (sequencing) | D | CH | Chorea. | Pulmonary infections. | NA |
| **Krude_2002_P4** | Adolescent | Frameshift *NKX2-1*(NM_?), (C2519A), p.? (sequencing) | NA | CH | Chorea. | NA | NA |
| **Krude_2002_P5** | Child | Frameshift *NKX2-1*(NM_?), (C1302A)/p.? (sequencing) | NA | CH | Muscular hypotonia. | Neonatal respiratory distress. | NA |
| **Li_2023_P1** | Child | Missense, *NKX2-1* (NM_001079668.3), c.706A>G/ p.Lys236Glu (WES) | D | CH | Dysarthria, ataxia, motor delay. | Pneumonia. | Language delay. |
| **Lynn_2020_P1** | Child | Deletion 14q13.1-14q21.1 (CGH array) | NA | CH | Muscular hypotonia. | Neonatal respiratory distress, pulmonary infections, pulmonary hypertension, tachypnoea, hypoxaemia, chronic respiratory failure. | Neurodevelopmental delay, feeding dysfunction. |
| **Magrinelli_2023_PII:2** | Adult | Insertion of 46-bp Alu sequence  chr14:36987132-GRCh37- (WES). | D | SH | Chorea, motor delay, cerebral palsy. | Neonatal respiratory distress, respiratory infections, dyspnoea. | Urinary infections, kyphoscoliosis. |
| **Makretskaya_2018_P74** | NA | Deletion, NKX2-1 (NM_001079668.3):chr14:36.986.917-36.987.061 or c.628_772del (NGS) | NA | CH | Chorea. | NA | NA |
| **Makretskaya_2018_P75** | NA | Missense, *NKX2-1* (NM_001079668.3), c.1180A>G/ p.Thr394Ala (NGS) | NA | CH | NA | NA | NA |
| **Maquet_2009_P1** | Child | Missense, *NKX2-1* (NM_001079668.3), c.709A>T/ p.Ile237Phe (sequencing) | D | CH (CpH later) | NA | Neonatal respiratory distress. | The patient passed away at 40 days (respiratory failure). |
| **Monti_2015_P1** | NA | Frameshift, *NKX2-1*(NM_001079668.3), c.390C>G/p. Tyr130Ter and splicing *NKX2-1*(NM_001079668.3),  c.463+41C>T/p.? (sequencing) | NA | CH | Chorea, muscular hypotonia. | NA | Developmental delay. |
| **Moya_2006_P1** | NA | Frameshift, *NKX2-1*(NM_001079668.3), c.915del /p.Ala306ArgfsTer75 (sequencing) | M | CH | Chorea, psychomotor delay. | NA | Memory and attention deficit. |
| **Moya_2006_P2** | NA | Frameshift, *NKX2-1*(NM_001079668.3), c.915del /p.Ala306ArgfsTer75 (sequencing) | M | CH | Chorea, loss of balance, tremor, hyperreflexia. | NA | Mental retardation. |
| **Moya_2006_P3** | NA | Frameshift, *NKX2-1*(NM_001079668.3), c.915del /p.Ala306ArgfsTer75 (sequencing) | D | Gestational H | NA | NA | NA |
| **Moya_2018_P1** | NA | Frameshift, *NKX2-1*(NM_001079668.3), c.223dupG/p.Val75Glyfs*334  (sequencing) | D | CH | Chorea, muscular hypotonia, psychomotor delay. | Tachypnoea, intercostal respiratory retractions, poor peripheral perfusion, cyanosis, lung diffuse ground glass opacity, hyaline membrane disease. | Flaring nostrils, gastroesophageal reflux. |
| **Nakamura_2012_P2 (III-3)** | NA | Frameshift, *NKX2-1*(NM_001079668.3), c.294C>G/ p.Tyr98Ter (sequencing) | D | Mild CH | Chorea, motor delay. | NA | NA |
| **Nakamura_2012_P1 (III-4) (proband)** | Adolescent | Frameshift, *NKX2-1*(NM_001079668.3), c.294C>G/ p.Tyr98Ter (sequencing) | D | Mild CH | Chorea, motor delay, | Pneumonia, asthma. | Intellectual disability. |
| **Nakamura_2012_P3 (II-4)** | NA | Frameshift, *NKX2-1*(NM_001079668.3), c.294C>G/ p.Tyr98Ter (sequencing) | D | H | Chorea, motor delay, involuntary movements,  muscle atrophy, and weakness. | NA | NA |
| **Narumi_2010_P1** | NA | Missense, *NKX2-1* (NM_001079668.3), c.268C>T / p.His90Tyr (sequencing) | NA | CH | NA | NA | NA |
| **Nattes_2017_P1** | Child | Frameshift, *NKX2-1*(NM_001079668.3), c.714G>A/ p.Trp238Ter (sequencing) | NA | H | Chorea, muscular hypotonia. | Neonatal respiratory distress. | NA |
| **Nattes_2017_P10** | Adult | Frameshift, *NKX2-1*(NM_001079668.3), c.267dup/ p.His90AlafsTer349  (sequencing) | NA | Gestational H | Chorea. | Infiltrative lung disease, lung fibrosis. | NA |
| **Nattes_2017_P11** | Adult | Splicing, *NKX2-1*(NM_001079668.3), c.463+2T>C/p.? (sequencing) | NA | H | Chorea. | Infiltrative lung disease, respiratory insufficiency | NA |
| **Nattes_2017_P14** | Adolescent | Deletion 14q13q13 (FISH) | NA | H | Chorea, Muscular hypotonia, psychomotor delay. | Neonatal respiratory distress. | NA |
| **Nattes_2017_P2** | Child | Frameshift, *NKX2-1*(NM_001079668.3), c.714G>A/p. Trp238Ter (sequencing) | NA | H | Muscular Hypotonia, psychomotor delay | Neonatal respiratory distress. | NA |
| **Nattes_2017_P3** | Child | Frameshift, *NKX2-1*(NM_001079668.3), c.344dup/ p.Tyr116LeufsTer323 (sequencing) | NA | H | Chorea, Muscular hypotonia, psychomotor delay. | Neonatal respiratory distress. | NA |
| **Nattes_2017_P4** | Child | Missense, NKX2-1 (NM_001079668.3), c.583C>T/p. Arg195Trp (sequencing) | NA | CpH | Muscular hypotonia. | Neonatal respiratory distress. | The patient passed away at 18 months (respiratory failure). |
| **Nattes_2017_P5** | Child | Frameshift, *NKX2-1*(NM_001079668.3), c.876_877del/ p.Leu293GlyfsTer145 (sequencing) | NA | H | Chorea, Muscular hypotonia, psychomotor delay. | Neonatal respiratory distress. | NA |
| **Nattes_2017_P6** | Child | Splicing, *NKX2-1*(NM_001079668.3), c.463+2T>C/p.? (sequencing) | NA | H | Chorea, Muscular hypotonia, psychomotor delay. | Neonatal respiratory distress. | NA |
| **Parnes_2019_P1** | Child | Frameshift, *NKX2-1*(NM_001079668.3),c.754_755insT /p.Asp252ValfsTer187 (WES) | D | H | Chorea, muscular hypotonia. | Neonatal respiratory distress, respiratory infections. | OCD, ADHD, developmental delay, stuttering. |
| **Parnes_2019_P2** | Child | Frameshift, *NKX2-1*(NM_001079668.3),c.390C>G / Tyr130Ter (WES) | M | H | Chorea, muscular hypotonia. | Neonatal respiratory distress, pulmonary hypertension. | Developmental delay. |
| **Parnes_2019_P3** | Adult | Frameshift, *NKX2-1*(NM_001079668.3),c.390C>G/ Tyr130Ter (WES) | D | H | Chorea, muscular hypotonia, dystonia, myoclonus. | Neonatal respiratory distress. | Developmental delay. |
| **Parnes_2019_P5** | Child | Frameshift, *NKX2-1*(NM_001079668.3),c.397dup/ p.Thr133AsnfsTer306 (sequencing) | NA | H | Muscular hypotonia, chorea. | NA | Epilepsy, developmental delay. |
| **Peall_2014_P10** | Adolescent | Missense, *NKX2-1*(NM_001079668.3), c.520G>T/p.Gly174Cys (sequencing) | D | SH | Chorea, muscular hypotonia, dystonia, ataxia. | NA | NA |
| **Peall_2014_P2** | Child | Frameshift, *NKX2-1*(NM_001079668.3), c.612del/ p.Tyr204Ter (sequencing) | P | CH | Muscular hypotonia, chorea, myoclonus, dysarthria. | Respiratory infections. | Developmental delay. |
| **Peall_2014_P3** | Child | Frameshift, *NKX2-1*(NM_001079668.3), c.428G>A / p.Trp143Ter  (sequencing) | M | CH | Chorea, muscular hypotonia, myoclonus, dystonia, dysarthria. | Neonatal respiratory distress. | Developmental delay. |
| **Peall_2014_P4** | Child | Whole gene deletion (sequencing) | D | CH, growth hormone deficiency. | Chorea muscular hypotonia. | Respiratory infections. | Developmental delay, visual impairment. |
| **Prasad_2019_P1** | Child | Deletion 14q13.2-q21.1 (35,975, 495–40,890, 854) (4,9Mb) Deletion 3p12.3-p13 73, (893, 402–74, 297, 269) (CGH array) | NA | CH, hyperinsulinism, growth hormone deficiency, cortisol deficiency, undetectable gonadotrophins, small pituitary. | Chorea. | Neonatal respiratory distress, pulmonary hypertension, ductus arteriosus, pulmonary infection. | NA |
| **Salerno_2014_P1** | Neonate | Frameshift, *NKX2-1*(NM_001079668.3), c.424G>T/ p.Gly142Ter (sequencing) | P | CH | Muscular hypotonia. | Neonatal respiratory distress, tachypnoea, ground glass areas in lungs. | NA |
| **Salvado_2013_P1** | Adolescent | Frameshift, *NKX2-1*(NM_001079668.3), c.915del/ p.Ala306ArgfsTer75 (NA) | M | CH | chorea, motor impairment, dyskinesia. | Neonatal respiratory distress. | Intellectual impairment. |
| **Salvado_2013_P2** | Adolescent | Frameshift, *NKX2-1*(NM_001079668.3), c.915del/ p.Ala306ArgfsTer75 (NA) | M | CH | Chorea, ataxia, dyskinesia, motor impairment. | Neonatal respiratory distress. | Intellectual impairment. |
| **Salvado_2013_P3** | Adult | Frameshift, *NKX2-1*(NM_001079668.3), c.915del/ p.Ala306ArgfsTer75 (NA) | M | Gestational H | Motor impairment. | NA | Poliomyelitis. |
| **Salvatore_2010_P1** | Adult | Frameshift, *NKX2-1*(NM_001079668.3), c.524C>A/ p.Ser175Ter (sequencing) | P | H | Chorea. | Ventricular dilatation. | Psychosis, memory deficit, brain hypometabolism. |
| **Salvatore_2010_P2** | Adult | Frameshift, *NKX2-1*(NM_001079668.3), c.524C>A/ p.Ser175Ter (sequencing) | NA | H | Chorea, hyperkinesia, apraxia, | Ventricular dilatation. | Development delay, memory deficit, brain hypometabolism. |
| **Salvatore_2010_P3** | Child | Frameshift, *NKX2-1*(NM_001079668.3), c.524C>A/ p.Ser175Ter (sequencing) | M | CH | Chorea. | Neonatal respiratory distress. | Developmental delay congenital anomalies (reflux with pyelectasis and megabladder, patent foramen ovale). |
| **Santos-Silva_2019_P10** | Child | Frameshift, *NKX2-1*(NM_001079668.3),c.859_860insTGCC/  p.Arg287Leufs*  (sequencing) | D | CH | Chorea, hypotonia, ataxia, dysmetria. | NA | Developmental delay. |
| **Shiohama_2018_P1** | Child | Deletion 14q13.2q13.34 (35,352,962–36,903,832)x1 dn (CGH array) | NA | Asymptomatic H | Chorea, muscular hypotonia, drop attacks. | NA | Developmental delay. |
| **Tanaka_2020_P26** | Child | Frameshift, *NKX2-1*(NM_001079668.3), c.596C>A/ p.Ser199Ter (sequencing) | NA | CH | NA | NA | NA |
| **Tozawa_2016_P1** | Child | Frameshift, *NKX2-1*(NM_001079668.3) c.915dup/p.Ala306ArgfsTer133 (sequencing) | NA | CH | Chorea, muscular hypotonia, ataxia, dystonia, cerebral palsy. | Neonatal respiratory distress, interstitial lung disease. | Developmental delay, ADHD. |
| **Trevisani_2022_P1** | Child | Frameshift, *NKX2-1*(NM_001079668.2) c.872C>G/Prol219ARg  (sequencing) | D | SH, growth hormone and serum insulin-like growth factor-I deficiency. | Hypotonia, dystonia, ligamentous hyperlaxity, developmental delay. | Asthma, tracheal-laryngomalacia, respiratory infections. | Otitis media, hippocampus dysmorphism. |
| **Uematsu_2012_P1** | Child | Deletion 14q12-13 (2.6 Mb) (CGH array) | D | CH | Chorea, gait disturbances, muscular hypotony. | NA | Developmental delay, reduced blood flow caudate nuclei. |
| **Uematsu_2012_P2** | Child | Missense, *NKX2-1*(NM_?), c.613G>T/  p.V205P (sequencing) | M | CH | Chorea, gait disturbances, muscular hypotony. | NA | Developmental delay, reduced blood flow caudate nuclei. |
| **Uematsu_2012_P3** | Child | Missense, *NKX2-1*(NM_?), c.613G>T/  p.V205P (sequencing) | M | H | Chorea, gait disturbances, muscular hypotony. | NA | Developmental delay, reduced blood flow caudate nuclei. |
| **Veneziano_2014_P II05 (proband)** | Adult | Frameshift, *NKX2-1*(ENST00000354822.7):c.631A>T/  p.Lys211Ter (sequencing) | D | H, macroadenoma, cystic pituitary mass. | Chorea, ataxia, dysarthria, dysdiadochokinesia. | NA | NA |
| **Veneziano_2014_P III01** | Adult | Frameshift, *NKX2-1*(ENST00000354822.7):c.631A>T/  p.Lys211Ter (sequencing) | M | H, low prolactin level, cystic pituitary mass. | Chorea, ataxia. | Chest infections | NA |
| **Villafuerte_2018_P1** | Child | Deletion14q13.2-q21.1 (3.32 Mb) (CGH array) | D | CH | Chorea, muscular Hypotonia, dysplasia, joint hyperlaxity. | Neonatal respiratory distress, respiratory infections. | Learning delay, immunodeficiency. supratentorial leukoencephalopathy. |
| **Villamil-Osorio_2021_P1** | NA | Deletion 14q12-q21.1 (14.7 Mb) (CGH array) | NA | H | NA | Lung sepsis, interstitial lung disease and hypertension. | Epilepsy, microcephaly, corpus callosum dysgenesis, supratentorial leukoencephalopathy. |
| **Williamson_2014_P1** | Adult | Missense, *NKX2-1* (ENST00000354822.7):c.626G>C/  p.Arg209Pro (sequencing) | D | CpH | Chorea, motor delay, ataxia, tremor. | NA | NA |
| **Williamson_2014_P2** | Adult | Missense, *NKX2-1* (ENST00000354822.7):c.626G>C/  p.Arg209Pro (sequencing) | D | CpH | Chorea. | NA | NA |
| **Williamson_2014_P3** | NA | Missense, *NKX2-1* (ENST00000354822.7):c.626G>C/  p.Arg209Pro (sequencing) | P | CpH | Chorea, motor delay, ataxia, tremor. | NA | NA |
| **Zou_2018_P28** | Child | NM_unknown, c.793_794insGGCGGCGGG/ p.? and c.760-886del127/ p.A254Tfs*85 (NGS) | D | CH | NA | NA | NA |

**Abbreviations:**

ADHD= attention-deficit/hyperactivity disorder

OCD= obsessive-compulsive disorder

**Age^a^.**

Neonate (≤ 28 days of life)

Child (≤12 years old)

Adolescent (13-19 years old)

Adult (20-65 years old)

NA=not available

**Gene test^b^.**

NKX2-1 gene sequencing

CGH= Comparative Genome Hybridization

FISH= fluorescence in situ hybridization

BAC = bacterial artificial chromosomes

NGS= Next-Generation DNA Sequencing

WES= Whole Exome DNA Sequencing

NA= not available

**Inherited^c^.**

D=de novo

M=maternal

P=paternal

NA= not available

**Endocrine alterations^d^**

H=hypothyroidism

CH=congenital hypothyroidism

SH=subclinical hypothyroidism

CpH=compensated hypothyroidism
