# Supplementary material for: Systematic review of thyroid function in NKX2-1-related disorders: Screening and diagnosis
Source: PLoS One. 2024 Jul 11;19(7):e0303880. doi: 10.1371/journal.pone.0303880 (PMC11238965; doi:10.1371/journal.pone.0303880)
Supplement: S5 File — Summary of thyroid pathology, hormone levels, and the results of screening and diagnostic tests for individual patients with NKX2-1-RD at the patient level. The table provides valuable insights into the specific thyroid-related abnormalities and hormone levels observed in each patient, along with the outcomes of screening and diagnostic procedures performed as part of the study. (DOCX) [file pone.0303880.s005.docx]

**S5. Summary of thyroid pathology, hormone levels, screening and diagnostic tests for patients with NKX2-1-related disorders. Patient level.**

| **Patient and reference** | **Thyroid alteration^a^** | **TSH neonatal**  **(mU/L)** | **FT4 neonatal**  **(pmo/L)** | **Hypothyroidism confirmation ^b^  (age at diagnosis)^c^** | **Diagnosis method** | **TSH at diagnosis**  **(mU/L)** | **FT4 at diagnosis**  **(pmol/L)** | **Thyroid gland alteration** |
| --- | --- | --- | --- | --- | --- | --- | --- | --- |
| **Balicza_2018_PII/2** | H | NA | NA | Y (adult) | Serum test. | High | NA | NA |
| **Barnett_2012_P1** | CH | normal | NA | Y (child) | Serum test. | 6.2 (NR 0.5-4.2) | Normal. | NA |
| **Barreiro_2011_P1** | CH | 8.3  (threshold 8.2) | NA | Y (neonate) | Serum test, scintigraphy, ultrasonography. | 126 (NR 0.3-5.5) | 9.65 (NR 1 - 24.1) | Small size. |
| **Carré_2009_P1** | Mild CH (CpH later) | NA | NA | Y (neonate) | Serum test, scintigraphy, ultrasonography. | 11 | 20 | Normal. |
| **Carré_2009_P2TA** | CH | NA | NA | Y (neonate) | Serum test, scintigraphy, ultrasonography. | 330 | 1.2 | Athyreosis. |
| **Carré_2009_P3TB** | CH | NA | NA | Y (neonate) | Serum test, scintigraphy, ultrasonography. | 31 | 13 | Hemiagenesis. |
| **Carré_2009_P4** | H | Normal | NA | Y (child) | Serum test, scintigraphy, ultrasonography. | 7.8 | 11 | Right lobe normal, left hypoplasia. |
| **Carré_2009_P5** | CH | NA | NA | Y (neonate) | Serum test, scintigraphy, ultrasonography, perchlorate discharge test. | 60 | 14 | Ectopic gland, normal size. |
| **Carré_2009_P6** | CH | NA | NA | Y (neonate) | Serum test, scintigraphy, ultrasonography. | 290 | 6 | Hypoplasia. |
| **Delestrain_2023_P2** | CH | NA | NA | Y (neonate) | Serum test, scintigraphy. | 420 (threshold <0.6) | 8  (NR 120-210) | Ectopic (sublingual localization). |
| **de Filippis_2014_P3** | CH | >100 (threshold <10) | NA | Y (neonate) | Serum test, scintigraphy, ultrasonography. | >200 (NR 0.4 – 6.3) | 2.57  (NR 22.9 – 54) | Ectopic. |
| **de Filippis_2014_P4** | Idiopathic Mild H | Normal (threshold <10) | NA | Y (child) | Serum test, ultrasonography. | 15.9 (NR 0.4 – 6.3) | 18 (NR 22.9- 54) | Normal. |
| **Doyle_2004_P I1** | H | NA | NA | Y (adult) | Serum test. | 20.3 | 99.11* | NA |
| **Doyle_2004_P II2** | H | NA | NA | Y (adolescent) | Serum test. | high | Normal * | NA |
| **Doyle_2004_P III2** | CH | 49 | Normal* | Y (neonate) | Serum test, scintigraphy, ultrasonography. | 17 | 108.12* | Right lobe normal, left decreased. |
| **Doyle_2004_P III3** | CH | 39 | Normal* | Y (neonate) | Serum test, scintigraphy, ultrasonography. | 24.5 | 81.09* | Right lobe normal, left decreased. |
| **Ferrara_2008_P1 (IV-1) (proband)** | CH | NA | NA | Y (neonate) | Serum test, scintigraphy. | NA | NA | No uptake in the neck. |
| **Ferrara_2008_P2 (III-1)** | H | NA | NA | Y (adult) | serum test, scintigraphy, thyroid ultrasonography. | 10.2 (NR 0.3–4.2) | 14.2  (NR 11.6-21.9) | Normal. |
| **Ferrara_2008_P3 (II-3)** | Mild H | NA | NA | Y (adult) | Serum test, ultrasonography. | 14.7 | 10.30 | Normal size. Hypoechogenic nodule in the right lobe. |
| **Gentile_2016_P1** | SH | NA | NA | Y (NA) | Serum test. | 19.7 (NR 0.4-3.7) | 13.27  (NR 9.8-18.8) | NA |
| **Gillett_2013_P1** | SH | 180700^#^ | NA | Y (neonate) | Serum test. | 93700^##^ | 14.2 | Normal. |
| **Gras_2012_P10_(D)** | CpH | NA | NA | Y (child) | Serum test. | NA | NA | NA |
| **Gras_2012_P11_(D)** | CpH | NA | NA | Y (child) | Serum test. | NA | NA | NA |
| **Gras_2012_P13_(E)** | CpH | NA | NA | Y (adult) | Serum test. | NA | NA | NA |
| **Gras_2012_P15_(E)** | CpH | NA | NA | Y (adult) | Serum test. | NA | NA | NA |
| **Gras_2012_P18_(E)** | CpH | NA | NA | Y (child) | Serum test. | NA | NA | NA |
| **Gras_2012_P19_(E)** | H | NA | NA | Y (adult) | Serum test. | NA | NA | NA |
| **Gras_2012_P2_(A)** | H | NA | NA | Y (adolescent) | Serum test. | NA | NA | NA |
| **Gras_2012_P20_(E)** | H | NA | NA | Y (adult) | Serum test. | NA | NA | NA |
| **Gras_2012_P21** | CpH | NA | NA | Y (child) | Serum test. | NA | NA | NA |
| **Gras_2012_P22** | H | NA | NA | Y (child) | Serum test. | NA | NA | NA |
| **Gras_2012_P23** | CH | NA | NA | Y (neonate) | Serum test. | NA | NA | NA |
| **Gras_2012_P24** | CH | NA | NA | Y (neonate) | Serum test. | NA | NA | NA |
| **Gras_2012_P26** | CH | NA | NA | Y (neonate) | Serum test. | NA | NA | NA |
| **Gras_2012_P27** | H | NA | NA | Y (child) | Serum test. | NA | NA | NA |
| **Gras_2012_P28** | CpH | NA | NA | Y (adolescent) | Serum test. | NA | NA | NA |
| **Gras_2012_P3_(A)** | H | NA | NA | Y (child) | Serum test. | NA | NA | NA |
| **Gras_2012_P4_(B)** | CpH | NA | NA | Y (child) | Serum test. | NA | NA | NA |
| **Gras_2012_P7_(B)** | CpH | NA | NA | NA (adult) | Serum test. | NA | NA | NA |
| **Hayasaka_2018_P7** | H | NA | NA | NA (NA) | NA | NA | NA | NA |
| **Hayasaka_2018_P8** | H | NA | NA | NA (NA) | NA | NA | NA | NA |
| **Hayasaka_2018_P9** | H | NA | NA | NA (NA) | NA | NA | NA | NA |
| **Hayashi_2015_PII1** | H | NA | NA | Y (NA) | Serum test. | NA | NA | NA |
| **Hayashi_2015_PII2 (proband)** | H | NA | NA | Y (neonate) | Serum test. | NA | NA | NA |
| **Hermanns_2018_P1** | CH | 49.5 (NR 0.4–9) | NA | Y (NA) | Serum test, ultrasonography. | NA | 48.4 *  (NR 50.2 - 196.9) | Hypoplasia. |
| **Kharbanda_2017_P1** | CH | 14 | NA | Y (neonate) | Serum test, scintigraphy, ultrasonography. | 25 (NR 0.7-13.1) | 15.5 (NR 9-26) | Hypoplasia. |
| **Kleinlein_2011_P1** | Mild CH | NA | NA | Y (neonate) | Serum test, ultrasonography. | 48 (threshold <24) | 18.4 (NR 11-22.3) | Normal. |
| **Koht_2016_P II:4** | H | NA | NA | NA (NA) | Serum test. | Normal. | Normal. | NA |
| **Koht_2016_P II:7** | H | NA | NA | NA (NA) | Serum test. | NA | NA | NA |
| **Koht_2016_P III:6 (index)** | CpH | NA | NA | NA (NA) | Serum test. | High. | Normal. | NA |
| **Koht_2016_P III:7** | CpH | NA | NA | NA (NA) | Serum test. | High. | Normal. | NA |
| **Koht_2016_P IV:3** | CpH | NA | NA | NA (NA) | Serum test. | High. | Normal. | NA |
| **Krude_2002_P1** | CH | NA | NA | Y (neonate) | Serum test, scintigraphy, ultrasonography. | 44 (NR 0.5-5) | 75.8 *  (NR 83.5-193.1) | Hypoplasia. |
| **Krude_2002_P2** | CH | NA | NA | Y (neonate) | Serum test, scintigraphy, ultrasonography. | 122 (NR 0.5-5) | 37.3 *  (NR 83.5-193.1) | Hypoplasia. |
| **Krude_2002_P3** | CH | NA | NA | Y (neonate) | Serum test, scintigraphy, ultrasonography. | 12 (NR 0.5-5) | 141.6 *  (NR 83.5-193.1) | Normal. |
| **Krude_2002_P4** | CH | NA | NA | Y (neonate) | Serum test, scintigraphy, ultrasonography. | 55 (NR 0.5-5) | 50.1 *  (NR 83.5-193.1) | Hypoplasia. |
| **Krude_2002_P5** | CH | NA | NA | Y (neonate) | Serum test, scintigraphy. | 825 (NR 0.5-5) | <12.8 *  (NR 83.5-193.1) | Agenesis. |
| **Li_2023_P1** | CH | 14.4 (threshold <10) | NA | Y (neonate) | Serum test, ultrasonography. | 36.3 (NR 0.3-5.5) | 11.9 (NR 11.2-22.6) | Small size. |
| **Lynn_2020_P1** | CH | normal | Low* | Y (neonate) | Serum test. | Normal. | Low. | NA |
| **Magrinelli_2023_PII:2** | SH | NA | NA | Y (adult) | Serum test. | NA | NA | NA |
| **Makretskaya_2018_P74** | CH | NA | NA | Y (NA) | Serum test. | > 90 | NA | Hypoplasia. |
| **Makretskaya_2018_P75** | CH | NA | NA | Y (NA) | Serum test. | > 90 | NA | Aplasia. |
| **Maquet_2009_P1** | CH (CpH later) | 31 (threshold <15) | 245 *  (NR 120–350) | Y (neonate) | Serum test. | 17.4 | 11.9 (NR 11–22.3) | Normal. |
| **Monti_2015_P1** | CH  (euthyrodi hypertireotropinemia at 8m) | 4.4 (threshold 10) | NA | Y (child) | Serum test, ultrasonography. | 8.8 (NR 0.5 – 5) | normal. | Normal. |
| **Moya_2006_P1** | CH | 186.2 (threshold 16.9) | 83.6 *  (threshold 123.6) | Y (neonate) | Serum test, scintigraphy. | High. | Low-normal* | Normotonic, bibulated gland with low uptake. |
| **Moya_2006_P2** | CH | 65.0 (threshold 14.3) | 124.8 * (threshold 273.8) | Y (neonate) | Serum test, scintigraphy. | High. | Low-normal* | normotonic, bibulated gland with low uptake. |
| **Moya_2006_P3** | gestational H | NA | NA | Y (NA) | Serum test. | Normal. | Normal. | NA |
| **Moya_2018_P1** | CH | 70 (threshold < 10) | NA | Y (neonate) | Serum test, scintigraphy, ultrasonography. | 224 | 7.7 | Small size. |
| **Nakamura_2012_P2 (III-3)** | mild CH | 12.6 | 14.68 | NA (NA) | serum test | NA | NA | NA |
| **Nakamura_2012_P1 (III-4) (proband)** | Mild CH | high | NA | Y (child) | Serum test, scintigraphy, ultrasonography. | 28.77 (NR 0.2-4) | 15.7 (NR 12.8 – 25.7) | Normal. |
| **Nakamura_2012_P3 (II-4)** | H | NA | NA | Y (adult) | Serum test. | 13.2 | 10.9 (NR 12.8-25.7) | NA |
| **Narumi_2010_P1** | CH | NA | NA | NA (NA) | NA | NA | NA | Normal. |
| **Nattes_2017_P1** | H | NA | NA | Y (child) | NA | NA | NA | NA |
| **Nattes_2017_P10** | Gestational H | NA | NA | Y (child) | NA | NA | NA | NA |
| **Nattes_2017_P11** | H | NA | NA | Y (child) | NA | NA | NA | NA |
| **Nattes_2017_P14** | H | NA | NA | Y (child) | NA | NA | NA | Normal. |
| **Nattes_2017_P2** | H | NA | NA | Y (child) | NA | NA | NA | Defect (no specification). |
| **Nattes_2017_P3** | H | NA | NA | Y (child) | NA | NA | NA | Ectopic. |
| **Nattes_2017_P4** | CpH | NA | NA | Y (child) | Serum test, ultrasonography. | 78 (threshold 15) | 12 (NR 11-22.3) | Normal. |
| **Nattes_2017_P5** | H | NA | NA | Y (child) | Serum test, ultrasonography. | 38 (threshold 15) | 14 (NR 11-22.3) | Normal. |
| **Nattes_2017_P6** | H | NA | NA | Y (child) | NA | NA | NA | Agenesis. |
| **Parnes_2019_P1** | H | NA | NA | Y (neonate) | NA | NA | NA | NA |
| **Parnes_2019_P2** | H | NA | NA | Y (neonate) | NA | NA | NA | NA |
| **Parnes_2019_P3** | H | NA | NA | Y (neonate) | NA | NA | NA | NA |
| **Parnes_2019_P5** | H | NA | NA | Y (neonate) | NA | NA | NA | NA |
| **Peall_2014_P10** | SH | NA | NA | Y (NA) | Serum test. | NA | NA | NA |
| **Peall_2014_P2** | CH | NA | NA | Y (NA) | Serum test. | NA | NA | NA |
| **Peall_2014_P3** | CH | NA | NA | Y (NA) | Serum test. | NA | NA | NA |
| **Peall_2014_P4** | CH | NA | NA | Y (NA) | Serum test. | NA | NA | NA |
| **Prasad_2019_P1** | CH | NA | NA | Y (neonate) | Serum test, ultrasonography. | 0.5 (NR 0.3-4.2) | 0.7 (NR 12-22) | NA |
| **Salerno_2014_P1** | CH | high | NA | Y (neonate) | Serum test, ultrasonography. | NA | NA | NA |
| **Salvado_2013_P1** | CH | NA | NA | Y (child) | Serum test. | High. | Normal. | Ectopic (sublingual). |
| **Salvado_2013_P2** | CH | normal | NA | Y (child) | Serum test. | NA | NA | Low uptake. |
| **Salvado_2013_P3** | Gestational H | NA | NA | NA (NA) | Serum test. | NA | NA | NA |
| **Salvatore_2010_P1** | H | NA | NA | Y (adult) | Serum test. | High. | NA | NA |
| **Salvatore_2010_P2** | H | NA | NA | Y (adult) | Serum test. | High. | NA | NA |
| **Salvatore_2010_P3** | CH | NA | NA | NA (NA) | Serum test. | NA | NA | NA |
| **Santos-Silva_2019_P10** | CH | NA | NA | NA (NA) | Serum test, ultrasonography. | NA | NA | Hypoplasia. |
| **Shiohama_2018_P1** | Asymptomatic  H | NA | NA | Y (child) | Serum test. | 10.9 (NR 0.3-4.9) | 14.2  (NR 9 – 19.1) | NA |
| **Tanaka_2020_P26** | CH | NA | NA | Y (NA) | Serum test, ultrasonography. | 29.6 (NR 0.3-4.2) | 9  (NR 10.3 – 20.6) | Normal. |
| **Tozawa_2016_P1** | CH | NA | NA | Y (neonate) | Serum test, ultrasonography. | 100 | 12.87 | Normal. |
| **Trevisani_2022_P1** | SH | NA | NA | Y (child) | Serum test. | 5.9 (NR 0.5-4.5) | 14.3 (NR 10.3-23.2) | NA |
| **Uematsu_2012_P1** | CH | NA | NA | Y (neonate) | Serum test. | NA | NA | NA |
| **Uematsu_2012_P2** | CH | NA | NA | Y (neonate) | Serum test. | NA | NA | NA |
| **Uematsu_2012_P3** | H | Normal | NA | Y (child) | Serum test. | NA | NA | NA |
| **Veneziano_2014_P II05 (proband)** | H | NA | NA | Y (adult) | Serum test. | NA | NA | NA |
| **Veneziano_2014_P III01** | H | NA | NA | Y (NA) | Serum test. | High. | Normal. | NA |
| **Villafuerte_2018_P1** | CH | 12.5 (threshold 10) | NA | Y (child) | Serum test, ultrasonography. | NA | NA | Hypoplasia (1.35ml, <P3 for age and sex). |
| **Villamil-Osorio_2021_P1** | H | NA | NA | NA (NA) | Serum test. | NA | NA | NA |
| **Williamson_2014_P1** | CpH | normal | NA | Y (child) | Serum test, perchlorate discharge test, hearing test. | High. | Normal. | Normal. |
| **Williamson_2014_P2** | CpH | normal | NA | Y (child) | Serum test. | 10 | Normal. | Normal. |
| **Williamson_2014_P3** | CpH | normal | NA | Y (neonate) | Serum test. | 7.3 | NA | Normal. |
| **Zou_2018_P28** | CH | NA | NA | Y (NA) | Serum test, scintigraphy, ultrasonography. | High. | Low. | Dysgenesis. |

**TSH:** thyroid stimulating hormone.

**FT4:** free thyroxine

**Values of hormone thyroid with the asterisk*=** total thyroxine (T4) measured in nmol/L.

**Values of hormone with the symbol^#^** = value was considered as 180.7 mU/L for calculations.

**Values of hormone with the symbol^##^** = value was considered as 93.7 mU/L for calculations.

**Endocrine alterations^a^**

H=hypothyroidism

CH=congenital hypothyroidism

SH=subclinical hypothyroidism

CpH=compensated hypothyroidism

**Hypothyroidism confirmation^b^**

Y=yes

NA=not available

**Age at diagnosis^c^.**

Neonate (≤ 28 days of life)

Child (≤12 years old)

Adolescent (13-19 years old)

Adult (20-65 years old)

NA=not available
