# Supplementary material for: Systematic review of thyroid function in NKX2-1-related disorders: Screening and diagnosis
Source: PLoS One. 2024 Jul 11;19(7):e0303880. doi: 10.1371/journal.pone.0303880 (PMC11238965; doi:10.1371/journal.pone.0303880)
Supplement: S6 File — The table displays the quality assessment of the included references concerning the detection and diagnosis of endocrine diseases in patients with NKX2-1-RD. Table A. Quality assessment of the case reports and case series. Table B. Quality assessment of the cohort studies. (DOCX) [file pone.0303880.s006.docx]

**S6. Quality assessment of included references for the detection and diagnosis of endocrine diseases in patients with NKX2-1-related disorders.**

**Table A. Quality of the care reports and case series included in this study**

|  |  | **Cases series /** | | **Case reports** | | | |  |  |  |
| --- | --- | --- | --- | --- | --- | --- | --- | --- | --- | --- |
|  | **Selection** | **Ascertainment** | | **Causality** | | | | **Reporting** | **Final score** | **Quality** |
| **Domain** | **1** | **2** | **3** | **4** | **5** | **6** | **7** | **8** |  |  |
| **Balicza_2018** | 0 | 0 | 1 | 0 | 0 | 0 | 1 | 0 | 2 | Poor |
| **Barnet_2012** | 0 | 0 | 1 | 0 | 0 | 0 | 1 | 0 | 2 | Poor |
| **Barreiro_2011** | 0 | 1 | 1 | 0 | 0 | 1 | 1 | 0 | 4 | Medium |
| **Carré_2009** | 0 | 0 | 1 | 0 | 0 | 0 | 1 | 0 | 2 | Poor |
| **De Filippis_2014** | 1 | 0 | 1 | 0 | 0 | 0 | 1 | 0 | 3 | Medium |
| **Delestrain_2023** | 0 | 0 | 1 | 0 | 0 | 0 | 1 | 0 | 2 | Poor |
| **Doyle_2004** | 0 | 0 | 1 | 0 | 0 | 0 | 1 | 0 | 2 | Poor |
| **Ferrara_2008** | 0 | 0 | 1 | 0 | 0 | 0 | 1 | 0 | 2 | Poor |
| **Gentile_2016** | 0 | 0 | 1 | 0 | 0 | 0 | 1 | 0 | 2 | Poor |
| **Gillett_2013** | 0 | 0 | 1 | 0 | 0 | 0 | 0 | 0 | 1 | Poor |
| **Gras_2012** | 0 | 0 | 1 | 0 | 0 | 0 | 1 | 0 | 2 | Poor |
| **Hayasaka_2018** | 1 | 0 | 1 | 0 | 0 | 0 | 0 | 0 | 2 | Poor |
| **Hayashi_2015** | 0 | 0 | 1 | 0 | 0 | 0 | 1 | 0 | 2 | Poor |
| **Hermanns_2018** | 1 | 1 | 1 | 0 | 0 | 0 | 1 | 0 | 4 | Medium |
| **Kharbanda_2017** | 0 | 1 | 1 | 0 | 0 | 1 | 1 | 0 | 4 | Medium |
| **Kleinlein_2010** | 0 | 0 | 1 | 0 | 0 | 0 | 1 | 0 | 2 | Poor |
| **Koht_2016** | 0 | 0 | 1 | 0 | 0 | 0 | 1 | 0 | 2 | Poor |
| **Krude_2022** | 0 | 0 | 1 | 0 | 0 | 0 | 1 | 0 | 2 | Poor |
| **Li_2023** | 0 | 0 | 1 | 0 | 0 | 0 | 1 | 0 | 2 | Poor |
| **Lynn_2020** | 0 | 0 | 1 | 0 | 0 | 0 | 1 | 0 | 2 | Poor |
| **Magrinelli_2023** | 0 | 0 | 1 | 0 | 0 | 0 | 1 | 0 | 2 | Poor |
| **Maquet_2009** | 0 | 0 | 1 | 0 | 0 | 0 | 0 | 0 | 1 | Poor |
| **Monti_2015** | 0 | 0 | 1 | 0 | 0 | 0 | 1 | 0 | 2 | Poor |
| **Moya_2006** | 0 | 0 | 1 | 0 | 0 | 0 | 1 | 0 | 2 | Poor |
| **Moya_2018** | 0 | 1 | 1 | 0 | 0 | 1 | 1 | 1 | 5 | Good |
| **Nakamura_2012** | 0 | 0 | 1 | 0 | 0 | 0 | 1 | 0 | 2 | Poor |
| **Nattes_2017** | 1 | 0 | 1 | 0 | 0 | 0 | 1 | 0 | 3 | Medium |
| **Parnes_2019** | 1 | 0 | 1 | 0 | 0 | 0 | 0 | 0 | 2 | Poor |
| **Peall_2014** | 1 | 0 | 1 | 0 | 0 | 0 | 1 | 0 | 3 | Medium |
| **Prasad_2019** | 0 | 0 | 1 | 0 | 0 | 0 | 1 | 0 | 2 | Poor |
| **Salerno_2014** | 0 | 0 | 1 | 0 | 0 | 0 | 0 | 0 | 1 | Poor |
| **Salvado_2013** | 0 | 0 | 1 | 0 | 0 | 0 | 1 | 0 | 2 | Poor |
| **Salvatore_2010** | 0 | 0 | 1 | 0 | 0 | 0 | 1 | 0 | 2 | Poor |
| **Santos-Silva_2019** | 1 | 0 | 1 | 0 | 0 | 0 | 1 | 0 | 3 | Medium |
| **Shiohama_2018** | 0 | 1 | 1 | 0 | 1 | 1 | 1 | 1 | 6 | Good |
| **Tanaka_2020** | 1 | 1 | 1 | 0 | 0 | 1 | 1 | 0 | 5 | Good |
| **Tozawa_2016** | 0 | 0 | 1 | 0 | 0 | 1 | 1 | 0 | 3 | Medium |
| **Trevisani_2022** | 0 | 1 | 1 | 0 | 0 | 1 | 1 | 0 | 4 | Medium |
| **Uematsu_2012** | 0 | 0 | 1 | 0 | 0 | 0 | 1 | 0 | 2 | Poor |
| **Veneziano_2014** | 0 | 0 | 1 | 0 | 0 | 0 | 1 | 0 | 2 | Poor |
| **Villafuerte_2018** | 0 | 0 | 1 | 0 | 0 | 0 | 1 | 0 | 2 | Poor |
| **Villamil-Osorio_2021** | 0 | 0 | 1 | 0 | 0 | 0 | 1 | 0 | 2 | Poor |
| **Williamson_2014** | 0 | 0 | 1 | 0 | 0 | 0 | 1 | 0 | 2 | Poor |
| **Zou_2018** | 0 | 0 | 1 | 0 | 0 | 0 | 0 | 0 | 1 | Poor |

|  | **Cohort studies** | | | |  |  | | |
| --- | --- | --- | --- | --- | --- | --- | --- | --- |
|  | **Selection** | | | | **Comparability** | **Outcome** | | |
| **Category** | **Representativeness of the exposed cohort** | **Selection of the non-exposed cohort** | **Ascertainment of exposure** | **Demonstration that outcome of interest was not present at start of study** | **Comparability of cohorts on the basis of the design or analysis** | **Assessment of outcome** | **Was follow-up long enough for outcomes to occur** | **Adequacy of follow up of cohorts** |
| **Makretskaya_2018** |  |  |  |  |  |  |  |  |
| **Narumi_2010** |  |  |  |  |  |  |  |  |

**Table B. quality of the cohort studies included in this study.**
